# PRDM12 Is Transcriptionally Active and Required for Nociceptor Function Throughout Life

**DOI:** 10.3389/fnmol.2021.720973

**Published:** 2021-09-27

**Authors:** Tomislav Kokotović, Michiel Langeslag, Ewelina M. Lenartowicz, John Manion, Christopher W. Fell, Elham Alehabib, Abbas Tafakhori, Hossein Darvish, Eric J. Bellefroid, G. Gregory Neely, Michaela Kress, Josef M. Penninger, Vanja Nagy

**Affiliations:** ^1^Ludwig Boltzmann Institute for Rare and Undiagnosed Diseases, Vienna, Austria; ^2^CeMM Research Center for Molecular Medicine of the Austrian Academy of Sciences, Vienna, Austria; ^3^Department of Neurology, Medical University of Vienna, Vienna, Austria; ^4^Department of Physiology and Medical Physics, Institute of Physiology, Medical University of Innsbruck, Innsbruck, Austria; ^5^Institute of Pharmacy and Center for Molecular Biosciences Innsbruck (CMBI), University of Innsbruck, Innsbruck, Austria; ^6^Department of Pharmacology, Medical University of Innsbruck, Innsbruck, Austria; ^7^Charles Perkins Centre, Dr. John and Anne Chong Lab for Functional Genomics, Centenary Institute, and School of Life and Environmental Sciences, University of Sydney, Camperdown, NSW, Australia; ^8^Student Research Committee, Department of Medical Genetics, School of Medicine, Shahid Beheshti University of Medical Sciences, Tehran, Iran; ^9^Iranian Center of Neurological Research, Neuroscience Institute, Tehran University of Medical Sciences, Tehran, Iran; ^10^Neuroscience Research Center, Faculty of Medicine, Golestan University of Medical Sciences, Gorgan, Iran; ^11^ULB Neuroscience Institute (UNI), Université Libre de Bruxelles (ULB), Gosselies, Belgium; ^12^Institute of Molecular Biotechnology of the Austrian Academy of Sciences, VBC – Vienna BioCenter, Vienna, Austria; ^13^Department of Medical Genetics, Life Science Institute, University of British Columbia, Vancouver, BC, Canada

**Keywords:** *Prdm12*, *TRPV1*, capsaicin, pain, nociception, CIP, dorsal root ganglia

## Abstract

PR domain-containing member 12 (PRDM12) is a key developmental transcription factor in sensory neuronal specification and survival. Patients with rare deleterious variants in *PRDM12* are born with congenital insensitivity to pain (CIP) due to the complete absence of a subtype of peripheral neurons that detect pain. In this paper, we report two additional CIP cases with a novel homozygous *PRDM12* variant. To elucidate the function of PRDM12 during mammalian development and adulthood, we generated temporal and spatial conditional mouse models. We find that PRDM12 is expressed throughout the adult nervous system. We observed that loss of PRDM12 during mid-sensory neurogenesis but not in the adult leads to reduced survival. Comparing cellular biophysical nociceptive properties in developmental and adult-onset PRDM12 deletion mouse models, we find that PRDM12 is necessary for proper nociceptive responses throughout life. However, we find that PRDM12 regulates distinct age-dependent transcriptional programs. Together, our results implicate PRDM12 as a viable therapeutic target for specific pain therapies even in adults.

## Introduction

Congenital insensitivity to pain (CIP), a subtype of hereditary sensory and autonomic neuropathies (HSAN), primarily affects the peripheral nervous system (PNS). Recently, mutations in methyltransferase PR domain-containing member 12 (PRDM12) have been demonstrated to cause CIP, which based on clinical symptoms was subtyped as HSAN type VIII (OMIM 616488) (Chen et al., 2015). PRDM12 belongs to a large family of transcription factors responsible for cell fate decisions (Hohenauer and Moore, 2012). At present, there are 24 reported patients with PRDM12-CIP, including the complete absence of all modalities of acute and chronic pain perception, frequently accidental or self-inflicted injuries, frequent bone fractures, the absence of corneal reflex and impaired tear production, and skin ulcerations and recurrent bacterial infections often requiring amputations. Unlike the association of CIP syndromes with other genes, such as nerve growth factor beta (*NGF*β), a very few PRDM12-CIP patients present with central nervous system (CNS) symptoms, such as intellectual disability (ID) (Chen et al., 2015; Imhof et al., 2020). Skin biopsies of patients revealed the absence of protein gene product 9.5- (PGP9.5) reactive nerve fiber endings in the epidermis, suggesting the participation of PRDM12 in nociceptor development or the survival in humans (Chen et al., 2015).

Nociceptors are pseudounipolar primary afferent sensory neurons in the PNS that detect noxious, potentially tissue-damaging, and painful stimuli. Their somata reside in the dorsal root ganglia (DRG) and trigeminal ganglion, with one axonal process in the periphery and one that synapses in the spinal cord (SC) or brain stem. Nociceptors express a variety of specialized noxious stimuli receptors for their function. These include transient receptor potential (TRP) cation channels, including TRPV1 and TRPA1 activated by capsaicin (Caterina et al., 1997) or noxious cold or electrophilic chemicals such as allyl isothiocyanate and cinnamaldehyde (Bandell et al., 2004; Julius, 2013). Additionally, potentially tissue-damaging temperatures are detected by a variety of voltage-gated Na^+^ channels, including Na_v_1.7, Na_v_1.8, and Na_v_1.9. Indeed, deleterious variants in genes encoding for Na_v_1.7 (*SCN9A*) and Na_v_1.9 (*SCN11A*) were also identified to be causative for a type of CIP (OMIM 243000 and OMIM 615548, respectively). Strong noxious stimulation of nociceptors will trigger afferent action potentials (APs) that send nociceptive signals to the CNS to induce the conscious sensation of pain.

PR domain-containing member 12 had no intrinsic enzymatic activity and was reported to recruit euchromatic histone-lysine N-methyltransferase 2 (EHMT2), also known as G9a, for the trimethylation of histone 3 lysine 9 (H3K9me3) resulting in subsequent regulation of target genes (Yang and Shinkai, 2013; Matsukawa et al., 2015). Detailed studies on *Danio rerio, Xenopus laevis*, and *Drosophila melanogaster* demonstrated that PRDM12 regulates a transcriptional program critical in cell fate decisions during early sensory neuronal development required for the specification and function of nociceptors (Matsukawa et al., 2015; Nagy et al., 2015; Thelie et al., 2015). Reduction of Hamlet, an ortholog of *PRDM12*, in *D. melanogaster* sensory neurons caused reduced nociceptive responses to noxious heat in fly embryos, a phenotype that could not be rescued by reconstitution with human *PRDM12* disease-causing variants, providing evidence of the contribution of variants to pathology (Nagy et al., 2015).

In mammals, embryonic mouse studies demonstrated that PRDM12 is expressed in DRGs and is necessary for the initiation and maintenance of an NGF-β receptor, neurotrophic receptor tyrosine kinase 1 (TrkA), by modulating neurogenins 1 and 2 (Ngn1 and Ngn2) activity, thereby promoting nociceptive lineage from neural crest cell progenitors (Kinameri et al., 2008; Bartesaghi et al., 2019; Desiderio et al., 2019). Correspondingly, the analysis of DRG from constitutive knock-out (KO, *Prdm12*^−/−^) mice revealed a significant reduction in DRG size, decreased density of cutaneous myelinated sensory branches and the absence of nociceptor-specific markers, all suggesting the absence of nociceptors (Bartesaghi et al., 2019; Desiderio et al., 2019). This specific reduction in nociceptor lineage seems to be a result of a proliferation defect during neurogenesis (Bartesaghi et al., 2019; Landy et al., 2021), however, an increase in apoptotic cell death with no change in proliferation was also reported (Bartesaghi et al., 2019; Desiderio et al., 2019). Constitutive embryonic PRDM12 deficiency resulted in early neonatal death suggesting that in mice, unlike humans, PRDM12 function is essential for survival (Bartesaghi et al., 2019; Desiderio et al., 2019).

Here, we aim to understand the functional role of PRDM12 in nociception in the mammal system. We first present two patients from a large consanguineous family with typical CIP symptoms, including the complete absence of pain perception, confirmed to have a previously unreported homozygous variant in *PRDM12*. To clarify the role played by PRDM12 in pain perception, we developed several different mouse models. To model human patient variants, we generated a mouse model with the previously reported CIP W160C PRDM12 single base-pair mutation (*Prdm12*^*W*160*C*^) and S159AfsTer2 (*Prdm12*^*S*159*AFsTer*2^) using clustered regularly interspaced short palindromic repeats- (CRISPR-) associated system 9 (Cas9) genome editing (Chen et al., 2015; Nagy et al., 2015). We additionally generated conditional mouse mutants, and therefore crossed *Prdm12*^*fl*/*fl*^ to *Sox10-Cre* (*Prdm12*^*fl*/*fl*^; *Sox10-Cre*) to ablate PRDM12 expression in the neural crest cell population during embryonic development, to *Avil-Cre* (*Prdm12*^*fl*/*fl*^; *Avil-Cre*) to ablate PRDM12 expression in the developing DRG and to tamoxifen- (TAM-) inducible *Rosa26-CreER*^*T*2^ mice (*Prdm12*^*fl*/*fl*^; *Rosa26-CreER*^*T*2^) to be able to induce whole-body PRDM12 KOs in adults. To combine genetic, cellular, electrophysiological, and molecular approaches, we were able to ascertain that PRDM12 is critical for survival and functional nociception in mice. Additionally, we were able to delineate the transcriptional program regulated by PRDM12, in developing and adult mammalian systems containing both core-PRDM12 regulated genes and those that are age-specific.

## Materials and Methods

### Patients and Whole-Exome Sequencing

Following informed consent and approval from patients and participating relatives, all procedures were performed. The procedures are in accordance with the ethical standards and approval of the Shahid Beheshti University of Medical Sciences ethics board and with the current update of the Declaration of Helsinki.

Whole-exome sequencing (WES) was performed at the Biomedical Sequencing Facility (BSF) in CeMM Center for Molecular Medicine of the Austrian Academy of Sciences (CeMM). Briefly, genomic DNA was extracted from the whole blood of patients, parents, and participating siblings using the QIAamp DNA Mini Kit (Qiagen, Hilden, Germany). The quantity and quality of DNA from index patient 1 were assessed by the Qubit 2.0 Fluorometric Quantitation system (Life Technologies, Carlsbad, CA, USA). Libraries were prepared using the Nextera DNA Flex Exome Library Prep Kit (Illumina, San Diego, CA, USA). Briefly, genomic DNA was tagmented, size-selected, and amplified followed by the two rounds of hybridization with biotinilated baits and capturing with streptavidin-conjugated magnetic beads. After enrichment, library fragments represented in the total 45 Mb coding region were amplified and size-selected. Final library pools were quality controlled and sequenced on a HiSeq 3000 instrument (Illumina, San Diego, CA, USA) using 75-bp paired-end chemistry. DNA sequences were mapped to GRCh37 (hg19) version of a human reference genome using a Burrows–Wheeler Aligner with default parameters. Single-nucleotide variants (SNVs) and indels were annotated with SNPEff (Cingolani et al., 2012), Combined Annotation Dependent Depletion (CADD; Kircher et al., 2014), the single-nucleotide polymorphism database (dbSNP; Sherry et al., 2001), the genome aggregation database (gnomAD; https://gnomad.broadinstitute.org/) (Karczewski et al., 2020), and ClinVar (Landrum et al., 2018) data. Subsequent filtering of the remaining variants of interest was based on a homozygous inheritance pattern, a variant type, the population frequency, and the gene lists of interest in relation to the symptoms of the patient. Further, the segregation analysis was performed in patient 2, as well as in both sets of parents and participating siblings by the conventional Sanger sequencing.

### Animals

*Prdm12*^*W*160^ experiments were approved by the University of Sydney Animal Ethics Committee under the animal ethics protocol (938), and the reporting complies with the animals in research: reporting *in vivo* experiments (ARRIVE) guidelines. Experimentation protocols comply with National Health and Medical Research Council (NHMRC) guidelines. Mice were housed in specific pathogen-free facilities at Australian BioResources, The Charles Perkins Centre and the Medical Foundation Building at the University of Sydney, Australia. *Prdm12*^*W*160^ mice were generated by CRISPR Cas9-mediated genome editing and were confirmed by the Sanger sequencing using the following primers: forward primer 5′-CTGCTTGGGAGTCTCTTAGAGAG-3′; reverse primer 5′-ATACAGCTGAACGAGGGTGA-3′; and sequencing primer (forward) 5′- TGACTCTCTATTGATCTTTTGCTTC-3′.

*Prdm12*^−/−^ or *Prdm12*^*fl*/*fl*^ mice were generated on C57BL/6 background using the promoter-driven knockout-first gene trap technique with En2SA-IRES-LacZ cassette in first intron and loxP sites around the second exon as described previously (Desiderio et al., 2019). Female mice carrying an conditional allele were crossed to *Advillin-Cre* or *Rosa26-Cre-ER*^*T*2^ male mice to generate corresponding developmental or TAM-inducible *Prdm12* conditional knockout offspring. Both *Prdm12*^*fl*/*fl*^; *Rosa26-CreER*^*T*2+^ and their *Prdm12*^*fl*/*fl*^*; Rosa26-CreER*^*T*2−^ littermates received 80 mg/kg intraperitoneal injections of TAM (Sigma-Aldrich, St. Louis, MO, USA) once per day for 4 consecutive days. TAM-injections were carried out at the age of 8 weeks, and animals were then tested 3 weeks following injections. TAM-injected *Prdm12*^*fl*/*fl*^; *Rosa26-CreER*^*T*2−^ animals are referred to as “controls.” These mice were housed at the Animal Facility of the Medical University of Vienna, and Comparative Medicine Mousehouse Vienna Biocenter, Vienna, Austria. All mice were maintained at a 12-h light/dark cycle and provided with food and water *ad libitum*. Experiments were approved by the Bundesministerium fur Wissenschaft, Forschung und Wirtschaft (BMWFW-66.015/0011-WF/V/3b/2017) and were carried out according to EU-directive 2010/63/EU. Genotyping was determined using the primers as follows: for *Cre*, forward primer 5′-GCTCGACCAGTTTAGTTACCC-3′, reverse primer 5′-TCGCGATTATCTTCTATATCTTCAG-3′; for *Prdm12* forward primer 5′-GCTGATCGAGTCCAGGAGAC-3′, reverse primer 5′-CCAAACATCCACAACCTTCA-3′. Select animals in each cohort were confirmed by qRT-PCR of DRG or whole brain (WB) tissue.

### Western Blotting

Tissue samples were collected in an RIPA (Sigma-Aldrich, St. Louis, MO, USA) buffer with Proteinase and Phosphatase inhibitor cocktail (Thermo Fischer Scientific, Waltham, MA USA) and homogenized using a Precellys 24 Tissue Homogenizer (Bertin Instruments, Frankfurt, Germany, 3 s × 20 s, 5,000 rpm). Protein concentration was measured using the Bradford assay (Bio-Rad, Hercules, CA, USA). Laemmli buffer- (Sigma-Aldrich, St. Louis, MO, USA) treated samples with standardized concentration of 1 μg/μl were incubated at 95°C for 5 min. About 25 μg of protein per lane was loaded on a 10% polyacrylic acid (PAA) gel, followed by protein transfer to a methanol-activated poly vinylidene fluoride (PVDF) membrane (GE Healthcare Life Sciences, Marlborough, MA, USA) for 10 h at 4°C with a constant current of 0.12 A. The membrane was then blocked with 5% bovine serum albumin (BSA) in TBS-T (0.01% Tween 20) for 1 h at room temperature (RT). Anti-PRDM12 (1:500, Santa Cruz, sc-130242) and loading control anti-HSP90α/β (1:1000, Santa Cruz, sc-13119) primary antibodies were incubated for 12 h at 4°C, followed by 1 h of incubation at RT with anti-mouse horseradish peroxidase- (HRP-) conjugated secondary antibody (1:30000, Sigma-Aldrich, St. Louis, MO, USA; GENA931). To control e specificity of the anti-PRDM12 antibody, human embryonic kidney (HEK) cell lysates overexpressing complementary DNA (cDNA) of human wild type (WT) PRDM12 (PRDM12^WT^) as a positive control or CIP-causing PRDM12 variant PRDM12^S58fs^ previously determined (Nagy et al., 2015) not to have a protein product, as a negative control were run alongside tissue lysates as labeled. The signal was visualized using a chemiluminescent reagent (ECL, GE Healthcare Life Sciences, Marlborough, MA, USA) and imaged using the ChemiDoc Imaging System (Bio-Rad, Hercules, CA, USA).

### Immunohistochemistry

Lumbar DRG from WT mice were dissected into ice cold phosphate-buffered saline (PBS), fixed in 4% formaldehyde for 20 min, and washed with PBS. Following cryopreservation in 30% sucrose, 20-μm sections were cut on a Microm Cryostat (HM560, Microm) at the Histopathology Service Facility in the Vienna Biocenter Core Facilities (VBCF), the member of the Vienna Biocenter (VBC), Austria, and stored in −80°C until needed. PBS hydrated tissue was permeabilized with 0.5% TritonX-PBS for 15 min and blocked for 1 h at RT with blocking buffer 10% normal goat serum in 0.25% TritonX-PBS. Following 2 × 3 min washes in 0.1% Tween20-PBS, the sections were incubated for 1 h at RT with goat F(ab) anti-mouse IgG (1:2000, Abcam, ab6668, Cambridge, UK) in 0.1% Tween20-PBS. Primary antibodies raised against IB4 (1:1000, Thermo Fisher, I21412, Waltham, MA, USA), Na_v_1.8 (1:200, Sigma-Aldrich Handels GmbH, Vienna, Austria, S2071), TrkA (1:200, GeneTex GTX54856, Irvine, CA, USA), or CGRP (1:500, Millipore, Burlington, MA, USA), with Guinea pig polyclonal anti-PRDM12 (1:10,000, clone 910) (Desiderio et al., 2019), diluted in a blocking buffer were incubated O/N at 4°C. Following 3 × 3 min washes in 0.01% TritonX-PBS, appropriate secondary antibodies (1:500 goat anti-mouse or anti-rabbit Alexa Fluor 488 or 546, Invitrogen, Waltham, MA, USA) diluted in 0.01% TritonX-PBS were incubated for 1 h at RT. Finally, following 3 × 3 min washes in 0.01% TritonX-PBS, the sections were mounted and imaged on the Zeiss LSM780 confocal microscope.

Skin biopsies were collected from hind paws, and flattened and fixed in 4% formaldehyde in PBS O/N at 4°C. Following cryopreservation in 30% sucrose, 20-μm sections were prepared as described above and stored at −80°C. Tissue was hydrated in PBS, incubated in 50 mM glycine/PBS for 1 h at RT, and blocked with 10% normal goat serum and 1% BSA in 0.1% TritonX-PBS. The sections were then incubated at 4°C O/N with a primary antibody raised against PGP9.5 (1:200, Zytomed 516-3344, Berlin, Germany) in blocking buffer. The sections were washed 3 × 3 min in 0.01% TritonX/PBS, followed by appropriate secondary antibody and nuclear label DAPI (1:2000, Carl Roth, Karlsruhe, Germany) in 0.01% TritonX-PBS and incubated for 1 h at RT. Following 3 × 3 min washes with 0.01% TritonX-PBS, the sections were mounted and imaged as mentioned earlier.

### Behavioral Assays

Behavioral assays were performed on both male and female mice at 8–15 weeks of age separated by at least 24 h between the tests to reduce handling stress. Experimenter was blind to the genotype of the test subjects.

*Intraplantar capsaicin injections*: about 1 μg of capsaicin (Sigma, St. Louis, MO, USA) diluted in 15 μl of normal saline was injected intraplantar in the hind paw of the animal. The animal was then observed for a total of 5 min, which is the time for the duration of reaction to the injection. Counted reactions included licking, shaking, or lifting of the injected paw. At the completion of 5 min, the animal was returned to its home cage. *Prdm12*^*fl*/*fl*^*; Avil-Cre*^+^ *N* = 33 was compared to the corresponding control littermates; and *N* = 26 of *Prdm12*^*fl*/*fl*^*; Rosa26-CreER*^*T*2+^ to *N* = 35 of their corresponding control littermates.

*Von frey test*: the measurements were performed according to Lau et al. (2019). Briefly, mice were habituated on 3 separate days and then tested three times with filaments ranging from 0.04 to 2 g with 10 applications per filament.

*The Hargreaves test*: mice were habituated on 3 separate days and then tested on 3 consecutive days with a low and a high input resistance (IR) intensity. The number of test animals in both von Frey and Hargraves assays was *N* = 16, compared to *N* = 18 of littermate controls.

*Open-field test* (*OFT*) was performed by the Preclinical Phenotyping Facility at the VBCF, the member of the Vienna Biocenter (pcPHENO, VBCF), Austria. Briefly, mice were placed in a 28 × 28 cm gray, plexi-glass arena and allowed to freely explore for 5 min. Time spent in the center or along the walls of the arena, as well as the distance traveled during exploration, were measured using an automated activity system (TSE-Systems, Bad Homburg, Germany). Reluctance to enter the center of the arena as compared to controls was scored and interpreted as the levels of anxiety. Distance traveled during the test was compared to controls and used as a readout for locomotion deficits.

*Morris water maze*: mice were trained in the Morris water maze (pcPHENO, VBCF) as described previously (Nagy et al., 2019). Briefly, mice were trained for two sessions a day, with four trials per session, using alternating entry points in different quadrants for each trial. Mice were video tracked using the software Topscan 3.0 (Cleversys, Inc., Reston, VA, USA). On day 1, the visual capacity of the mice was checked by making the platform visible with a black flag and letting the mice explore the pool for 1 min or until they reached the platform (data not shown). Short-term memory test was performed after the last trial, on days 8 and 11 to test for long-term memory. The time spent on searching the target quadrant and target zone (exact location of the platform) was recorded and used as a readout for memory. For both, OFT and Morris water maze *N* = 8 of *Prdm12*^*fl*/*fl*^*; Rosa26-CreER*^*T*2+^ were compared to *N* = 12 of their corresponding control littermates.

### Micro CT Imaging

For microscopic x-ray imaging, samples were contrasted with IKI (Lugol's iodine solution) (Metscher, 2009) and mounted in an aqueous medium (Metscher, 2011). MicroCT images were acquired using the Xradia MicroXCT system (www.zeiss.com/microscopy/int/x-ray.html) in the Theoretical Biology Department at the University of Vienna. Scans were made with the source settings of 60 kVp at 133 μA and source-sample-detector distances chosen to optimize the field of view for the sample. Tomographic slices were reconstructed using the Xradia XMReconstructor software and saved as TIFF image stacks with an isotropic voxel size of 7.5 μm. The segmentation and processing of images were performed manually using Amira Software 6.4.

### Single-Cell Electrophysiology

*Sensory neuron culture*: lumbar DRG were harvested from adult mice (age > 8 weeks) as discussed in previous publications (Langeslag et al., 2014; Namer et al., 2017). The sensory neurons were plated on glass coverslips coated with poly-L-lysine/laminin-1 (Sigma-Aldrich, Merck, Burlington, MA, USA) and cultivated in supplemented neruronal culture media (TNB) (Biochrom) containing 25 ng/ml mNGF 2.5S (Alomone Labs, Jerusalem, Israel) at 37°C and 5% CO_2_ in a humidified incubator for 16–24 h.

Cultured sensory neurons were used for electrophysiological experiments 16–24 h after seeding. Only DRG neurons with size smaller than <35 μm were selected, representing the small- to medium-sized neurons. Glass coverslips were mounted in a recording chamber and placed on a Zeiss Axiovert 200 microscope. All measurements were recorded with an EPC 10 and the Patchmaster v2.73 software (HEKA) at RT. For more details on the methodology for single-cell recordings, please see Supplementary Materials.

From the isolated sensory neurons, cellular voltage and current recordings were performed in a whole-cell patch-clamp configuration. The DRG neurons were kept in extracellular solution (ECS) containing (in mM); NaCl (150), KCl (5), CaCl_2_ (2), MgCl_2_ (1), HEPES (10), glucose (10), and the pH was set to 7.3 with NaOH. Borosilicate glass pipettes (Science Products) were pulled with a horizontal puller (P-97, Sutter Instrument Company, Novato, CA, USA) and filled with intracellular solution (ICS) containing (in mM); K-gluconate (98), KCl (50), CaCl_2_ (0.5), MgCl_2_ (2), EGTA (5), HEPES (10), MgATP (2), NaGTP (0.2), and the pH was adjusted to 7.3 with KOH.

In current-clamp recordings, the recorded neurons were kept at the holding current of 0 pA. The IR of sensory neurons was determined by four increasing hyperpolarizing current injections (Δ-5 pA from a holding current of 0 pA, 5 kHz).

The minimal current to evoke a single AP within 50 ms (I_AP_) for each neuron was obtained by 5 pA stepwise increasing, depolarizing instanteous, block pulses, and APs were recorded at 20 kHz. The amplitudes of 5 s ramp-shaped depolarizations were set at 1x, 2x, and 3x I_AP_ and sampled at 5 kHz. The amplitude of the 20 s depolarizing pulse was set to 2x I_AP_ for the corresponding neuron, and the voltage changes were recorded at 5 kHz.

A seven-barrel system with the common outlet was used for the heat stimulation of single neurons (Dittert et al., 1998). In voltage clamp recordings, the neurons were clamped at a holding potential of −60 mV. Heat-activated inward currents (I_Heat_) were elicited by applying ramp-shaped heat stimuli at 60 s intervals (linear temperature increases from RT to 50°C within 5 s). Neurons were assigned as heat responsive when they showed a non-linear inward current with a threshold below −0.2 nA from the baseline current.

### Electrophysiological Analysis

*Action potential analysis*: the average IR was calculated according to Ohm's law. From the AP evoked by injecting depolarizing current pulses (50 ms, sampled at 20 kHz), the resting membrane potential (*V*_mem_), afterhyperpolarization (AHP), and overshoot (OS) of *Prdm12*^*fl*/*fl*^*; Avil-Cre*^+^ and *Prdm12*^*fl*/*fl*^*; Avil-Cre*^−^ DRG neurons were determined. From the first derivative of the evoked APs, the maximal speeds of depolarization (S1) and of the biphasic repolarization (S2 and S3) were derived. Further, the time between S1, S2, and/or S3 (t1-t2; t2-t3; and t1-t3) was determined to compare AP width. The corresponding membrane voltage was deducted at the point where the falling slope of the 1st derivative reversed into a rising slope, and the AP threshold was determined (Langeslag et al., 2014; Namer et al., 2017).

### RNA Sequencing and Real-Time qPCR

*RNA isolation*: lumbar DRG tissues were dissected from 10 to 14-week-old animals, immediately placed in RNALater solution (Ambion, Austin, TX, USA), and snap frozen in liquid nitrogen until further isolation. Tissues were lysed using Precellys 24 Tissue Homogenizer (Bertin Instruments, Paris, France) 3×30 s at 5000 rpm, by QIAshredder (Qiagen, Valencia, CA, USA) homogenization. RNA was isolated using the RNeasy Mini Kit (Qiagen, Valencia, CA, USA) with on-column DNease treatment with RNase-Free Dnase Set (Qiagen, Valencia, CA, USA). A minimum of three biological replicates per genotype were used.

*Real-time q-PCR*: reverse transcription was performed using the qScript cDNA Synthesis Kit (Quantabio, Beverly, MA, USA) with an input amount of 200 ng of RNA, as per the instructions of the manufacturer. Real-time quantitative PCRs (RT-qPCRs) were done using an iTaq Universal SYBR Green Supermix (Bio Rad, Los Angeles, CA, USA) and run on a StepOnePlus Real-Time PCR System (Applied Biosystems, Darmstadt, Germany). The comparative Ct method (ΔΔCt) was used to determine relative gene expression, normalizing to GAPDH. The primers used for qPCR are *Gapdh* forward: 5'-GGTCGTATTGGGCGCCTGGTCACC-3'; reverse: 5'-CACACCCATGACGAACATGGGGGC-3'; *Prdm12* (exon 3) forward: 5'-CTACATCAAGTGTGCCCGGA−3'; reverse: 5'- TGGCCTTGTAGAAGATGCTCG-3'.

*RNA sequencing*: the amount of RNA was quantified using a Qubit 2.0 Fluorometric Quantitation System (Life Technologies, Carlsbad, CA, USA), and an Experion Automated Electrophoresis System (Bio-Rad, Hercules, CA, USA) was used to calculate the RNA integrity score. RNA-sequencing (RNA-seq) libraries were prepared using the TruSeq Stranded messenger RNA (mRNA) LT sample preparation kit Illumina, San Diego, CA, USA) using Sciclone and Zephyr liquid handling robotics (PerkinElmer, Spokane, WA, USA) at pre- and post-PCR steps. Library concentrations were determined using a Qubit 2.0 Fluorometric Quantitation system (Life Technologies, Carlsbad, CA, USA), and the distribution of sizes was determined using an Experion Automated Electrophoresis System (Bio-Rad, Hercules, CA, USA). For sequencing, the 14 libraries were pooled, diluted to equimolar amounts, and sequenced on an Illumina HiSeq 3000/4000 using 50-bp single-end chemistry. Base calls provided by the real-time analysis (RTA) software (Illumina, San Diego, CA, USA) were converted into a multiplexed, unaligned BAM format before demultiplexing into sample-specific unaligned BAM files.

### Transcriptome Analysis

Next generation sequencing (NGS) samples (passing quality filtering) were aligned to the UCSC Genome Browser (*Mus musculus* genome assembly mm10, Mus musculus transcriptome mm10_e100) of the genome reference consortium (GrCh38/GrCm38) using the STAR alignment tool (Dobin et al., 2013). The Bioconductor DESeq2 package (Love et al., 2014) was used for testing differentially expressed genes (DEGs) using the negative binomial distribution.

### Transcriptome Enrichment Analysis

*Overrepresentation analysis* of the unranked list of significantly (FDR < 0.05) DEGs common to both animal models was performed. The analysis utilized g:profiler package (version e103_eg50_p1) (Reimand et al., 2007), specifically g:GOSt Functional profiling tool with the Benjamini-Hochberg FDR threshold cutoff of 0.05. Gene sets utilized in analysis included gene ontology molecular function (GO:MF) and Kyoto Encyclopedia of Genes and Genomes (KEGG) pathway sets. The 10 most enriched terms for both GO:MF and KEGG pathways were represented in a decreasing order using –log10(FDR) value.

*Gene set enrichment analysis (GSEA)* was performed using the GSEA software. As GSEA input expression data set values, DeSeq2 normalized counts were used. The analysis performed for MSigDB gene sets (Subramanian et al., 2005; Liberzon et al., 2011) from C2: KEGG pathways, C5: GO:MF, and cutoffs for the value of *p* < 0.05 and FDR < 0.25. GO:MF gene sets were further imported into Cytoscape (v.3.8.2) (Shannon et al., 2003), and the pathways were clustered into networks using EnrichmentMap (v.3.3.1) (Merico et al., 2010). For network generation (Reimand et al., 2019), a similarity cutoff of 0.375 for combined Overlap+Jaccard score and combined score of 0.5 was used.

### Data-Analyses and Statistics

All electrophysiological data were analyzed using the Origin software (Originlab, Origin Pro 8, North Hampton, NH, USA). Statistical analysis (Graphpad Prism 7.0, GraphPad Software Inc., San Diego, CA) was performed by either the Mann–Whitney test in case of non-normal-distributed data or *t*-test for normal-distributed data (Shapiro–Wilk). The distribution of heat-responsive sensory neurons was tested using a Fisher's exact test. Statistical analysis of behavioral data and qPCR was performed by unpaired Student's *t*-test (capsaicin injections, Von Frey assay, Hargreaves test, and OFT, qRT-PCR). For Morris water maze, unpaired Student's *t*-test was used to analyze the short- and long-term memory data and two-way ANOVA with Sidak's multiple comparisons test for the latency to reach the platform.

## Results

### Identification of Novel Splice-Site Variant in Two Related Cases of CIP

**Figure 1:**
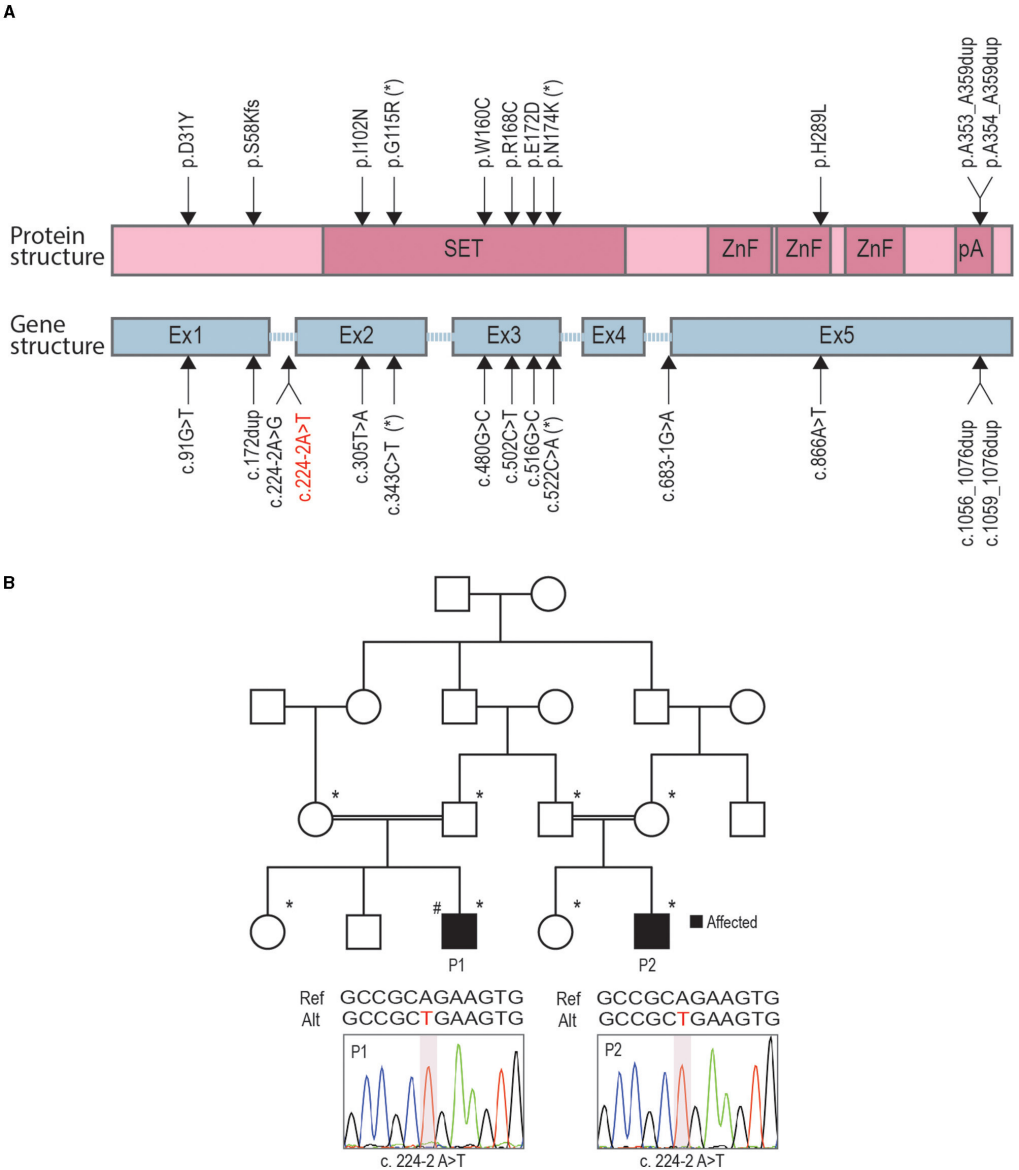
Identification of novel variants in two related congenital insensitivity to pain (CIP) cases. **(A)** A schematic representation of PR-domain containing member 12 (PRDM12) protein domain structure (pink) and gene structure (blue) with marked locations of previously published causative CIP variants and a novel splice-site variant reported here (red letters). **(B)** Family pedigree, with affected individuals shaded in black; family members included in segregation analysis indicated with asterisks; hashtag denotes index patient (patient 1, P1) for which whole-exome sequencing (WES) was performed. The position of the missense mutation is depicted below the family pedigree for patients 1 (P1) and 2 (P2) with an illustrative Sanger chromatogram of genomic PCR amplicons.

Currently, there are 24 known PRDM12-CIP patients from different ethnic backgrounds (Figure 1A). To contribute a better understanding of prevalence and the contribution of a PRDM12 variant to nociceptive dysfunction, we present two cases harboring an unreported *PRDM12* variant. Two affected first-order male cousins from an Iranian Farsi consanguineous family presented to the clinics with the absence of acute and chronic pain perception starting from birth (Figure 1B, Table 1). There is no history of CIP in the family. Index patient [patient 1 (P1)] was first presented with a tongue ulceration, and was later confirmed to have disturbed temperature and vibration sensation, sensorineural deafness, corneal abrasions, frequent skin and bone infections, and scoliosis. Patient 2 (P2) exhibited very similar symptoms with a few minor differences (Table 1). While both patients were determined to exhibit autism spectrum disorder (ASD), ID, and muscular weakness, patient 1 also presented with mild cortical malformation as evaluated by MRI (data not shown). Due to clinical similarity to other PRDM12-CIP cases, the targeted Sanger sequencing was performed on the DNA sample from patient 1 that revealed a homozygous splice acceptor site variant within intron 1 of *PRDM12* NM_021619:c.224-2 A>T (chr9: 133541993) (Figure 1B). Considering that ASD and ID are not commonly associated with PRDM12-CIP, and to rule out any other known CIP-causing gene contribution, we performed WES on the DNA sample from patient 1. WES analysis confirmed the presence of the *PRDM12* variant with a CADD score (Kircher et al., 2014) of 29.4. Subsequently, we confirmed that the variant segregated with the disease in a recessive fashion: in a homozygous state in patients 1 and 2 and in a heterozygous state in each of the four parents (Figure 1B). A different homozygous variant in the same position (c.224-2A>G) has previously been published in a patient suffering from CIP accompanied with global developmental delay, similarly to the patients we describe (Saini et al., 2017). Subsequent filtering of the WES failed to detect any additional rare, high CADD-PHRED scoring variants within the other known CIP genes, including *NGF, NTRK1, SCN9A, SCN11A, ZFHX2, FAAHP1*, and *CLTCL1*. To identify potential variants contributing to the autism and ID phenotype, we performed a search for rare variants in the genes associated with ASDs and ID as per PanelApp corresponding panels (Martin et al., 2019). The analysis identified rare variants within nine genes confirmed or predicted to be causative for ADD and/or ID in patient 1, however, subsequent Sanger sequencing of these loci in patients 1 and 2 revealed that none were shared (Table 2).

**Table 1:** Clinical synopsis of patients 1 and 2.

**Patient**		**P1**	**P2**
Ethnic origin		Farsi	Farsi
Sex		M	M
Age		19	9
Parental consanguinity		4th degree consanguinity (first cousins)	4th degree consanguinity (first cousins)
Gene		*PRDM12*	*PRDM12*
Variant zygosity		Homozygous	Homozygous
Genomic change (GRCh37)		chr9:g.133541993A>T	chr9:g.133541993A>T
Variant cDNA position		ENST00000253008.2:c.224-2A>T	ENST00000253008.2:c.224-2A>T
Age of onset		At birth	At birth
First sign		Tongue ulceration	Tongue ulceration
Pain sensitivity	Acute	Absent	Absent
	Chronic	Absent	Absent
	Inflammatory	Absent	Absent
	Segmental or global	Global pain insensitivity	Global pain insensitivity
Thermosensitivity		Impaired	Impaired
Vibration		Impaired	Impaired
Proprioception		N/A	Yes
Touch		N/A	Yes
Corneal ulcerations		Yes	Yes
Hypo-/alacrimia		Yes	Yes
Smell		N/A	Hyposmia
Hearing		Sensineural deafness	Sensineural deafness
Deep tendon reflexes		Trace	Trace
Motor weakness		Mild	Mild
Life threatening hyperthermia		N/A	No
Intellectual disability		Moderate	Mild
Autism		Yes	Yes
Behavioral stereotypy		Yes	No
Brain MRI findings		Mild cortical malformation	No
Sensory nerve conduction		Impaired	Impaired
Ulcers and mutilation		Oral cavity, lips	Oral cavity, lips
Frequent skin infections		Yes	Yes
Bone infections		Yes	No
Other phenotypes		Hypertensia, scoliosis, hypersalivation	Hypertension, scoliosis

**Table 2:** Rare homozygous variants in autism and/or intellectual disability- (ID-) associated genes identified in patient 1 whole-exome sequencing (WES).

**Gene**	**Zygosity**	**Genomic change (GRCh37)**	**P1 variant** **genotype**	**P2 variant** **genotype**	**PanelApp panel**	**OMIM gene entry**	**Inheritance**	**Segregation result**
UNC80	Homozygous	chr2:g.210678477C>T	T/T	C/T	ID	612636	AR	Excluded
POLA2	Homozygous	chr11:g.65063046T>C	C/C	T/C	Autism candidate	N/A	N/A	Excluded
KCNK7	Homozygous	chr11:g.65363204C>T	T/T	C/T	Autism candidate	603940	N/A	Excluded
CEP290	Homozygous	chr12:g.88500631C>A	A/A	C/A	ID	610142	AR	Excluded
ACE	Homozygous	chr17:g.61566077G>C	C/C	G/G	Autism candidate	106180	N/A	Excluded
ARFGEF2	Homozygous	chr20:g.47632862A>G	G/G	A/G	ID	605371	AR	Excluded
PLA2G6	Homozygous	chr22:g.38508527C>T	T/T	C/C	ID	603604	AR	Excluded
BCOR	Homozygous	chrX:g.39932734G>C	C	G	ID	300485	XLD	Excluded
BRWD3	Homozygous	chrX:g.79938123T>C	T	C	ID	300553	XLR	Excluded

*N/A, inheritance mode unknown*.

### PRDM12 Is Expressed in the Mouse Nervous System Through Life

**Figure 2:**
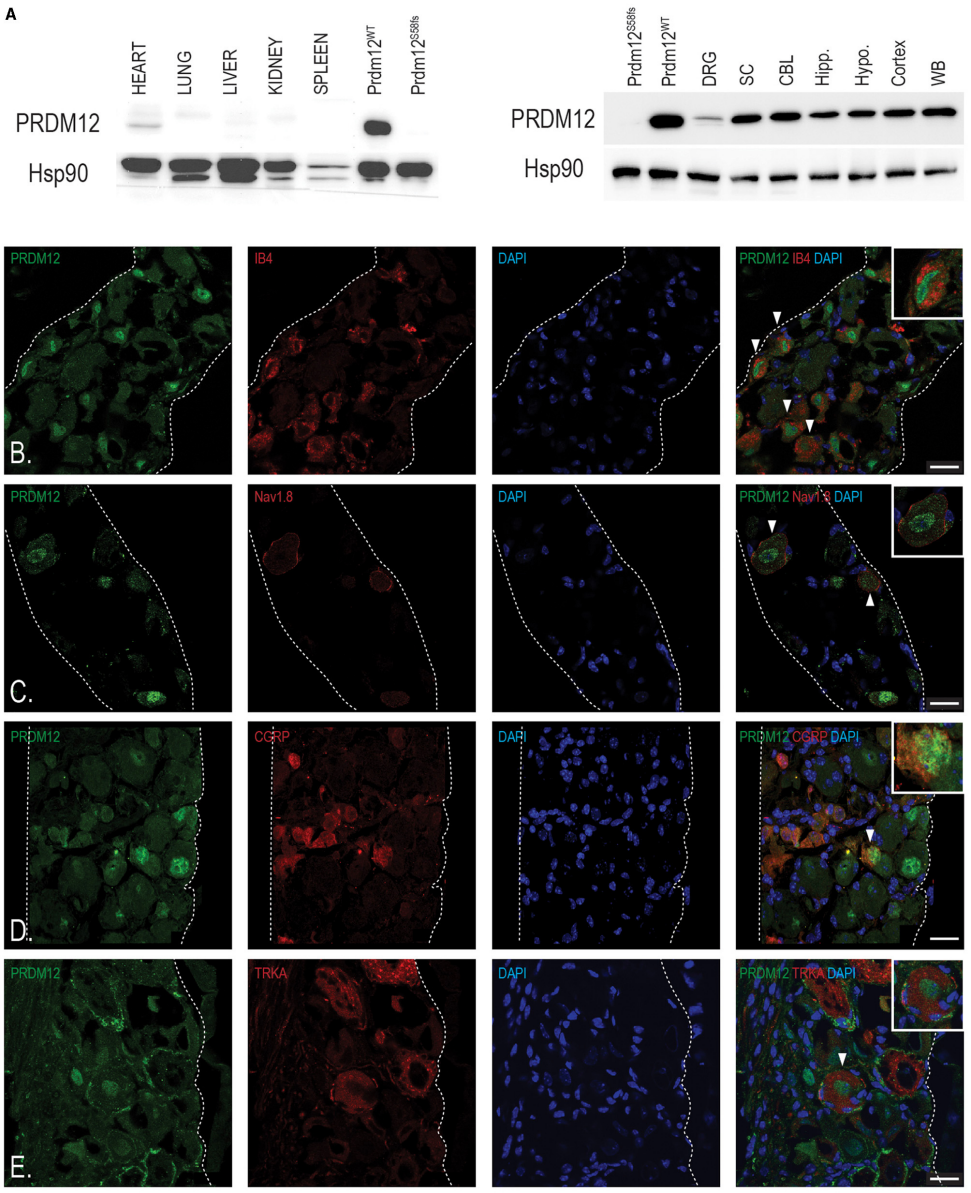
Tissue analyses reveal PRDM12 expression in adult mouse peripheral and central nervous tissue. **(A)** Upper panel, western blot analysis shows a slight PRDM12 protein expression in adult heart tissue, but no detectable expression in the lungs, livers, kidneys, or spleen. Lower panel, western blot indicates PRDM12 protein level expression in the central nervous system (CNS): whole brain (WB), cortex, hypothalamus (Hypo.), hippocampus (Hipp.), cerebellum (CBL), and spinal cord (SC), as well as in peripheral nervous system (PNS), and dorsal root ganglia (DRG). Antibody positive control (PRDM12^WT^) is HEK293 cell lysate overexpressing wild type (WT) PRDM12 complementary DNA (cDNA) and negative control (PRDM12^S58fs^) is HEK203 cell lysate overexpressing PRDM12^S58fs^ cDNA. HSP90 was used as loading control. **(B–E)** Representative immunohistochemical images of adult mouse lumbar DRG sections indicating PRDM12 co-expression with classical nociceptive markers: IB4, Na_v_1.8, CGRP, and tyrosine kinase 1 (TRKA), as labeled. White arrows indicate cells with co-labeling, with digitally enlarged insets of select co-labeled cells. Scale bar in all images is 20 μm.

To understand PRDM12 expression pattern in the mouse, we performed the western blotting analysis of various tissues harvested from adult WT mice. HEK293 cell lysates overexpressing either WT PRDM12 (PRDM12^WT^) or previously published frameshift mutation *Prdm12*^S58fs^ (PRDM12^S58fs^) that leads to no protein product (Nagy et al., 2015) were blotted to ensure antibody specificity (Figure 2A). In addition, low expression in the heart, we did not detect PRDM12 immunoreactivity in any other non-nervous tissue we examined (Figure 2A, upper panel). We detected PRDM12 immunoreactivity in both peripheral and CNS tissue samples (Figure 2A, lower panel). Interestingly, PRDM12 expression was noted in all brain regions examined. As previously reported in embryonic tissue, immunohistochemical double labeling of adult WT DRG sections revealed PRDM12 to remain co-expressed with various polymodal nociceptive markers, including IB4, Na_v_1.8, CGRP, and TRKA (Figures 2B–E) (Desiderio et al., 2019).

### PRDM12 Loss During Embryogenesis but Not in Adult Mice Leads to Reduced Survival

**Figure 3:**
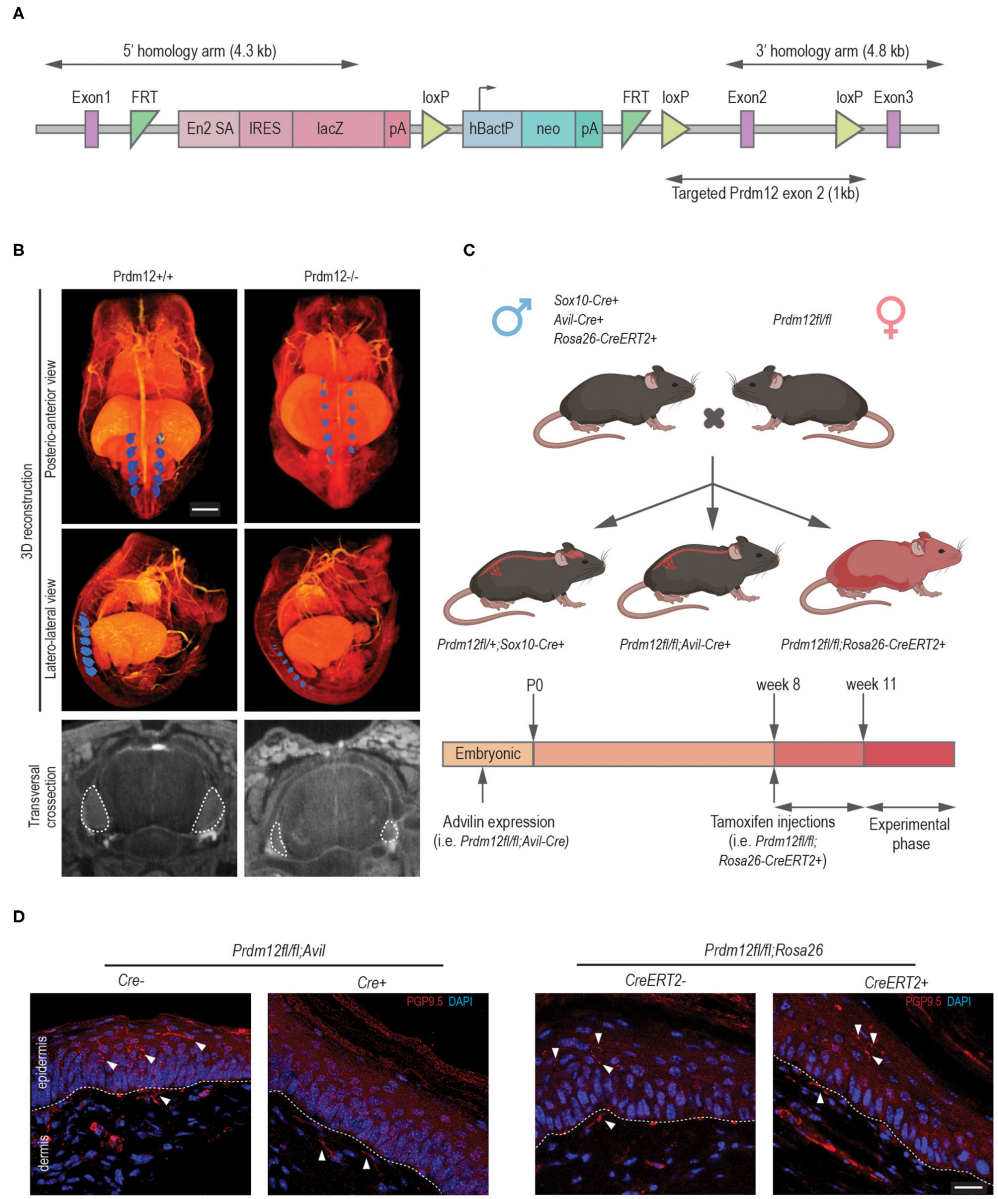
Validation of animal models generated for the study. **(A)** A schematic representation of the lethal classical *Prdm12* knock-out (KO) allele. **(B)** Lower panels, representative lumbar DRG cross-section micro-CT images of WT and *Prdm12*^−/−^ E15.5 embryos depicting a reduction of DRG volume in the *Prdm12*^−/−^. Middle and upper panels—pseudocolored (blue) 3D reconstructed micro-CT images of lumbar DRG in WT and *Prdm12*^−/−^ 15.5 embryos, as indicated—posteroanterior (upper panels) and latero-lateral view (middle panels). Scale bar is 1,000 μm. Transversal cross-section of the SC with DRG outlined with a white dotted line (bottom panel). **(C)** Schematic of the genetic crosses performed to generate developmental constitutive *Prdm12*^*fl*/*fl*^; *Avil-Cre* and *Prdm12*^*fl*/*fl*^; *Sox10-Cre*, and inducible *Prdm12*^*fl*/*fl*^; *Rosa26-Cre*ER^T2^, with the timeline of tamoxifen (TAM) injection, analysis or tissue harvest below. **(D)** Left two panels: representative immunohistochemical images of hind paw skin biopsy from *PRDM12*^*fl*/*fl*^*; Avil-Cre*^+^ show the complete absence of PGP9.5-reactive nerve fibers in the epidermal layer as compared to the *Cre*^−^ controls. In contrast, PGP9.5-reactive nerve fibers are clearly visible in the epidermal layers in representative images of TAM-injected *Prdm12*^*fl*/*fl*^; *Rosa26-CreER*^*T*2+^ hind paw skin biopsy (right two panels) as compared to the *Cre*^−^ controls. Dotted line delineates epidermis/dermis border. Scale bar is 20 μm.

Constitutive, “classic” KO (Figure 3A) of *Prdm12* (*Prdm12*^−/−^) results in a significant reduction of DRG volume (Figure 3B) and early neonatal death of unknown cause (Desiderio et al., 2019). We were, therefore, motivated to generate a mouse model that more closely resembles human disease as most CIP-causing variants of *PRDM12* do not affect its protein expression (Nagy et al., 2015). Therefore, using the Cas9 methodology, we generated a frameshift at position c.537A_del (p.S159AfsTer2; *Prdm12*^*S*159*AfsTer*2^) and point mutation at c.542G>C (p.W160C; *Prdm12*^*W*160*C*^) in murine *Prdm12* gene (NM_001123362, Supplementary Figure 1A) to reflect the previously reported PRDM12-CIP mutation W160C (Nagy et al., 2015). To improve viability, mice were maintained on a mixed FVB/NJ/C57/BL6 background. *Prdm12*^*S*159*AfsTer*2^ mutation is predicted to cause an early stop codon and likely mimic the constitutive *Prdm12*^−/−^ absence of PRDM12 protein expression. Both *Prdm12*^*S*159*AfsTer*2^ and *Prdm12*^*W*160*C*^ mice, similarly to the constitutive *Prdm12*^−/−^, showed complete penetrance of prenatal lethality in homozygous mutants. Heterozygous *Prdm12*^*S*159*AfsTer*2+/−^ mice had normal pain perception as compared to WT littermates when tested in acute pain assays (Supplementary Figure 1B).

To understand the functional role of PRDM12, we generated several conditional *Prdm12* mutant mouse models. We aimed to determine if PRDM12 function was necessary for pain perception when ablated during early development and/or in adults. We first crossed *Prdm12*^*fl*/*fl*^ to *Sox10-Cre* (*Prdm12*^*fl*/*fl*^; *Sox10-Cre*), thereby eliminating PRDM12 expression in the entire neural crest cell population during embryonic development (Figure 3C). Notably, we did not detect any viable *Prdm12*^*fl*/*fl*^; *Sox10-Cre*^+^ offspring. Thermal and chemical acute pain tests (hot plate assay and capsaicin intraplantar injection, respectively) on heterozygous *Prdm12*^fl/+^; *Sox10-Cre*^+^ did not reveal any reduction in pain perception when compared to control littermates (data not shown).

We next sought to ablate PRDM12 expression specifically in the DRG, thus we crossed *Prdm12*^*fl*/*fl*^ to *Avil-Cre* (*Prdm12*^*fl*/*fl*^; *Avil-Cre*) to remove PRDM12 expression approximately at embryonic day 12.5 (Figure 3C) (Hasegawa et al., 2007; Desiderio et al., 2019). RT-qPCR and the western blotting of lumbar DRG tissue confirmed a significant reduction of Prdm12 transcript and protein expression, respectively (Supplementary Figure 2A). We noted a reduction of the survival of *Prdm12*^*fl*/*fl*^*; Avil-Cre*^+^ animals (Supplementary Figure 2B). The animals that died did so before weaning for reasons that have yet to be determined. In *Prdm12*^*fl*/*fl*^; *Avil-Cre*^+^ survivors, we noted corneal abrasions and facial scarring similar to PRDM12-CIP patients (Supplementary Figure 2C) (Desiderio et al., 2019; Chen et al., 2020).

To delete PRDM12 in adults, we crossed *Prdm12*^*fl*/*fl*^ to TAM-inducible *Rosa26-CreER*^*T*2^ mice (*Prdm12*^*fl*/*fl*^; *Rosa26-CreER*^*T*2^). Following TAM injections at 8 weeks of age, we generated whole-body PRDM12 KOs in adults 3 weeks following injection as confirmed by RT-qPCR in lumbar DRG and WB tissue (Figure 3C, Supplementary Figure 3A). Unlike the constitutive (*Prdm12*^−/−^) or neuronal crest cell progenitor PRDM12 KOs (*Prdm12*^*fl*/*fl*^; *Sox10-Cre*^+^), there was no noted reduction in the survival of adult TAM-injected *Prdm12*^*fl*/*fl*^; *Rosa26-CreER*^*T*2+^ as compared to control littermates. As PRDM12 is expressed in the mouse brain, we aimed to identify any confounding CNS effects that may have resulted by the reduction of PRDM12 expression in the CNS. OFT of TAM-injected *Prdm12*^*fl*/*fl*^; *Rosa26-CreER*^*T*2+^ revealed no deficiencies in the distance traveled or increased anxiety as compared to control littermates (Supplementary Figure 3B). Furthermore, the Morris water maze test and the gold standard for hippocampal-dependent spatial learning, revealed no deficiencies in swimming ability, learning acquisition, or short- or long-term memory retention (Supplementary Figure 3C).

To determine the effect of nociceptor development in our conditional mouse models and the extent to which they phenocopy the symptoms of patients, we performed the immunohistochemical analysis of hind paw skin biopsies from both *Prdm12*^*fl*/*fl*^; *Avil-Cre* and TAM-injected *Prdm12*^*fl*/*fl*^; *Rosa26-CreER*^*T*2^ adult mice with anti-PGP9.5 antibody, a marker for small caliber, intraepidermal nerve fibers. PGP9.5 immunostaining is considered as a diagnostic tool in the study of peripheral neuropathies, which, in PRDM12-CIP case, revealed the absence of PGP9.5 reactive nerve endings in patient epidermis (Chen et al., 2015; Van Acker et al., 2016). As it was noted for PRDM12-CIP patients, *Prdm12*^*fl*/*fl*^*; Avil-Cre*^+^ biopsies had no detectable PGP9.5 reactive fibers in the epidermis, in contrast to control littermates (Figure 3D). TAM-injected *Prdm12*^*fl*/*fl*^; *Rosa26-CreER*^*T*2+^, on the other hand, had normal epidermal PGP9.5^+^ innervation (Figure 3D).

### PRDM12 Expression Is Required for Functional Nociceptors in Cultured DRG

To investigate the functional nociceptive role of PRDM12 in the two mouse models at a cellular level, we performed single-cell electrophysiological studies on cultured DRG. We isolated DRG from *Prdm12*^*fl*/*fl*^*; Avil-Cre*^+^ and TAM-injected *Prdm12*^*fl*/*fl*^*; Rosa26-CreER*^*T*2+^ and their respective controls and cultured them for ~16 h. The resting membrane potential (*V*_mem_) of *Prdm12*^*fl*/*fl*^*; Avil-Cre*^+^ DRG neurons was similar to *Prdm12*^*fl*/*fl*^*; Avil-Cre*^−^ DRG neurons in whole-cell current-clamp recordings (Table 3). However, the IR in the *Prdm12*^*fl*/*fl*^*; Avil-Cre*^+^ DRG neurons was significantly low. Consequently, the minimal current to evoke an AP within 50 ms (I_AP_) was increased to more than two times in *Prdm12*^*fl*/*fl*^*; Avil-Cre*^+^ neurons. The ablation of the *Prdm12* gene after TAM-injection in *Prdm12*^*fl*/*fl*^; *Rosa26-CreER*^*T*2^ DRG neurons, however, showed no differences in the resting membrane potential, IR and I_AP_ compared to DRG neurons were derived from control, TAM-injected *Prdm12*^*fl*/*fl*^; *Rosa26-CreER*^*T*2−^, mice (Table 3).

**Table 3:** Action potential (AP) characteristics of *Prdm12^fl/fl^*; *Avil-Cre^+^* and *Prdm12^fl/fl^*; *Rosa26-CreER^T2+^* nociceptive neurons and corresponding controls.

	***Prdm12 ^fl/fl^***; ***Avil-Cre^−^*** **(*N* = 32, *n* = 3)**	***Prdm12 ^fl/fl^***; ***Avil-Cre^+^*** **(*N* = 38, *n* = 3)**		***Prdm12^fl/fl^***; ***Avil-CreER^T2−^*** **(*N* = 37, *n* = 4)**	***Prdm12^fl/fl^***; ***Avil-CreER^T2+^*** **(*N* = 29, *n* = 3)**	
**Action potential**						
Input Resistance (GΩ)	1.53 ± 0.14	0.81 ± 0.11	^***^	1.33 ± 0.11	1.54 ± 0.18	n.s.
I_AP_ (pA)	69.84 ± 12.6	162.4 ± 20.9	^***^	47.16 ± 7.36	50.17 ± 11.9	n.s.
V_mem_ (mV)	−56.28 ± 1.42	−56.29 ± 0.90	n.s.	−51.34 ± 1.17	−52.52 ± 1.29	n.s.
OS (mV)	68.97 ± 0.52	49.89 ± 1.84	^***^	56.97 ± 0.54	56.90 ± 0.46	n.s.
AHP (mV)	−66.66 ± 0.81	−66.66 ± 0.48	n.s.	−66.71 ± 0.60	−66.30 ± 0.94	n.s.
AP Treshold (mV)	−28.98 ± 0.71	−30.89 ± 1.39	n.s.	−28.29 ± 0.92	−27.09 ± 0.88	n.s.
S1 (mV.ms^−1^)	127.64 ± 3.79	116.94 ± 5.44	n.s.	73.92 ± 2.63	72.63 ± 2.95	n.s.
S2 (mV.ms-^1^)	−38.80 ± 2.20	−92.89 ± 4.72	^***^	−20.95 ± 1.09	−20.10 ± 1.32	n.s.
S3 (mV.ms^−1^)	−30.87 ± 1.83	−73.63 ± 4.91	^***^	−27.23 ± 1.40	−23.91 ± 1.50	n.s.
t1-t2 (ms)	1.66 ± 0.10	0.79 ± 0.02	^***^	1.93 ± 0.06	2.17 ± 0.08	n.s.
t2-t3 (ms)	5.87 ± 0.98	1.51 ± 0.12	^***^	5.51 ± 0.21	5.88 ± 0.27	n.s.
t1-t3 (ms)	4.21 ± 0.89	0.70 ± 0.10	^***^	3.58 ± 0.18	3.70 ± 0.21	n.s.
**Rheo ramp**						
1xIAP	0.67 ± 0.36	5.09 ± 1.05	^***^	2.97 ± 0.88	6.24 ± 1.19	^**^
2xIAP	3.58 ± 1.57	13.71 ± 1.81	^***^	10.5 ± 1.75	14.65 ± 2.37	n.s.
3xIAP	7.78 ± 2.94	22.31 ± 2.60	^***^	18.41 ± 2.78	22.41 ± 3.26	n.s.
**2xIAP Rheo20s**						
0.0–2.5s	7.5 ± 2.15	11.25 ± 1.61	n.s.	9.41 ± 1.69	13.24 ± 2.05	^*^
2.5–5.0s	3.20 ± 1.48	13.06 ± 2.05	^***^	9.52 ± 1.87	13.44 ± 2.40	n.s.
5.0–7.5s	2.20 ± 1.15	15.21 ± 2.47	^***^	10.35 ± 1.91	14.20 ± 2.52	n.s.
7.5–10.0s	1.58 ± 0.82	16.75 ± 2.67	^***^	10.29 ± 1.78	13.58 ± 2.24	n.s.
10.0–12.5s	1.02 ± 0.59	16.09 ± 2.55	^***^	10.94 ± 1.76	13.72 ± 2.30	n.s.
12.5–15.0s	0.88 ± 0.53	14.28 ± 2.42	^***^	11.97 ± 1.86	13.96 ± 2.37	n.s.
15.0–17.5s	0.91 ± 0.60	12.96 ± 2.39	^***^	11.91 ± 1.80	16.06 ± 2.69	n.s.
17.5–20.0s	1.02 ± 0.72	12.06 ± 2.30	^***^	11.94 ± 1.75	16.48 ± 2.69	n.s.
Total #APs	16.35 ± 6.10	110.58 ± 16.9	^***^	86.35 ± 13.2	114.72 ± 15.3	n.s.

*I_AP_, minimal current to evoke a single AP within 50 ms; V_mem_, resting membrane potential; OS, overshoot; AHP, afterhyperpolarization; S1, maximal speed of depolarization; S2 and S3, biphasic repolarization speeds; n.s, not significant.*

* *p > 0.05,*

** *p > 0.01,*

*** *p > 0.001, N, number of cells; n, number of mice*.

**Figure 4:**
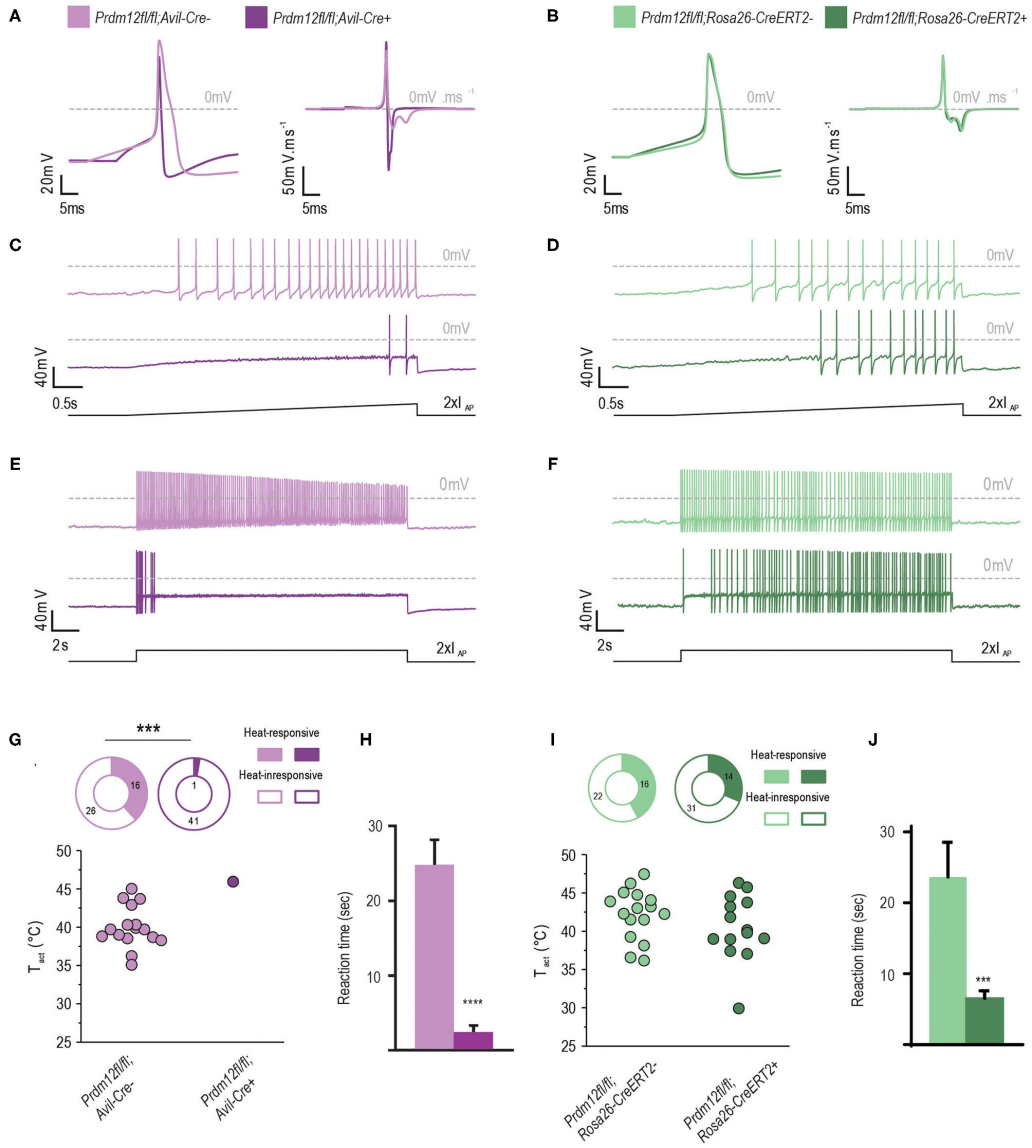
PRDM12 expression is required for proper biophysical properties of cultured DRG. **(A)** and **(B)** Representative action potentials (APs) and their first derivatives from *Prdm12*^*fl*/*fl*^*; Avil-Cre*^+^ [**(A)**, dark purple trace], *Prdm12*^*fl*/*fl*^*; Avil-Cre*^−^ [**(A)**, light purple trace], *Prdm12*^*fl*/*fl*^; *Rosa26-CreER*^*T*2+^ [**(B)**, dark green trace], and *Prdm12*^*fl*/*fl*^; *Rosa26-CreER*^*T*2−^ [**(B)**, light green trace] cultured DRG neurons. Note the reduced AP width and lack of biphasic repolarization in *Prdm12*^*fl*/*fl*^*; Avil-Cre*^+^ **(A)**. **(C,D)** Typical examples of AP generation upon 2 x I_AP_ ramp-shaped depolarization showing the inability of *Prdm12*^*fl*/*fl*^*; Avil-Cre*^+^ DRG neurons to generate similar number of APs compared to the *Prdm12*^*fl*/*fl*^*; Avil-Cre*^−^ DRG neurons **(C)**. Note the delay of AP firing for initial 2 s in the ramp-shaped depolarization in *Prdm12*^*fl*/*fl*^*; Avil-CreER*^*T*2+^, as compared to littermate controls **(D)**. **(E)** *Prdm12*^*fl*/*fl*^*; Avil-Cre*^+^ neurons dot not generate APs over the full time course of 2 x I_AP_ current injection compared to *Prdm12*^*fl*/*fl*^*; Avil-Cre*^−^ neurons. **(F)** The TAM-injected *Prdm12*^*fl*/*fl*^*; Rosa26-CreER*^*T*2+^ and *Prdm12*^*fl*/*fl*^*; Rosa26-CreER*^*T*2−^ generate APs over the full length of 2 x I_AP_ current injection although the TAM-injected *Prdm12*^*fl*/*fl*^*; Rosa26-CreER*^*T*2+^ show a reduced number of APs in the beginning of the 2 x I_AP_ current injection. **(G,I)** Fraction of neurons that showed temperature-evoked currents (top panels) and temperature activation thresholds (*T*_act_) of the recorded neurons (bottom panels). *Prdm12*^*fl*/*fl*^*; Avil-Cre*^+^ neurons do not respond to the applied ramp-shaped temperature increase **(G)**, whereas in both fractions of TAM-injected *Prdm12*^*fl*/*fl*^; *Rosa26-CreER*^*T*2+^ and *Prdm12*^*fl*/*fl*^*; Rosa26-CreER*^*T*2−^ neurons their respective T_act_ were similar **(I)**. **(H)** *Prdm12*^*fl*/*fl*^*; Avil-Cre*^+^ exhibited reduced behavioral reactions to intraplantar capsaicin injections as compared to their respective littermate controls (*N* = 33, mean values 12.38 ± 1.702 SEM vs. *N* = 18 1.216 ± 0.4454 SEM). **(J)** TAM-injected *Prdm12*^*fl*/*fl*^*; Rosa26-CreER*^*T*2+^ exhibited reduced behavioral reactions to intraplantar capsaicin injections as compared to their respective littermate controls (*N* = 26, mean values 23.66 ± 4.887 SEM vs. *N* = 35 6.555 ± 1.04 SEM). Values of ***p ≤ 0.005, ****p ≤ 0.0001, ns: not significant. Lower black traces are examples of the applied stimulus protocol.

To investigate the properties of APs between the corresponding genotypes, we injected the currents at IAP + 10 pA of the recorded neurons. Remarkably, APs evoked from *Prdm12*^*fl*/*fl*^*; Avil-Cre*^+^ were significantly different from those derived from *Prdm12*^*fl*/*fl*^*; Avil-Cre*^−^. The AP width recorded from cultured *Prdm12*^*fl*/*fl*^*; Avil-Cre*^+^ DRG neurons was significantly smaller than those from *Prdm12*^*fl*/*fl*^*; Avil-Cre*^−^ neurons and seem to lack the typical biphasic repolarization phase (also known as “hump” or “shoulder”), which is a characteristic of nociceptive DRG (Figure 4A) (Ritter and Mendell, 1992). This reduction in AP width (t1–t3, Table 3) in *Prdm12*^*fl*/*fl*^*; Avil-Cre*^+^ is correlated with increased repolarization speeds (d*V*/d*t* min_1_ and d*V*/d*t* min_2_, Table 3), whereas the depolarization speed (d*V*/d*t* max) is unchanged. Additionally, the AP peak (OS) was significantly lower in *Prdm12*^*fl*/*fl*^*; Avil-Cre*^+^ DRG neurons, whereas the AHP was unchanged (Figure 4A, Table 3). The AP threshold did not differ between *Prdm12*^*fl*/*fl*^*; Avil-Cre*^−^ and *Prdm12*^*fl*/*fl*^*; Avil-Cre*^+^ DRG neurons. The AP characteristics *V*_mem_, IR, and I_AP_ recorded from *Prdm12*^*fl*/*fl*^; *Rosa26-CreER*^*T*2+^ and *Prdm12*^*fl*/*fl*^; *Rosa26-CreER*^*T*2−^-derived neurons have not shown any differences (Figure 4B, Table 3).

We applied two different depolarization protocols to evaluate the excitability of the DRG neurons. During a ramp-shaped depolarization, the *Prdm12*^*fl*/*fl*^*; Avil-Cre*^+^ neurons generated fewer APs (Figures 4C,D, Table 3). Additionally, the number of APs generated in a 20-s long, block-shaped depolarization by *Prdm12*^*fl*/*fl*^*; Avil-Cre*^+^ neurons were significantly low (Figure 4E, Table 3) and the *Prdm12*^*fl*/*fl*^*; Avil-Cre*^+^ neurons predominantly fired within the seconds of the depolarization (Table 3). In contrast to *Prdm12*^*fl*/*fl*^*; Avil-Cre*^+^, the *Prdm12*^*fl*/*fl*;^*Avil-Cre*^−^ neurons generated more APs during ramp-shaped depolarizations and constantly during the long block-shaped depolarization. Meanwhile, there were no differences observed in the AP characteristics between TAM-injected *Prdm12*^*fl*/*fl*^*; Rosa26-CreER*^*T*2+^ and *Prdm12*^*fl*/*fl*^*; Rosa26-CreER*^*T*2−^ neurons, both excitability protocols showed a minor difference between the two genotypes. In the 1 × I_AP_ depolarizing ramp, the *Prdm12*^*fl*/*fl*^; *Rosa26-CreER*^*T*2+^ showed a reduced number of Aps, and additionally, in the prolonged 2× I_AP_ depolarization, they showed a reduced number of APs within the first few seconds (Figure 4F, Table 3).

Non-specific activation of Na+ channels by prepulse at −120 mV revealed larger currents in *Prdm12*^*fl*/*fl*^*; Avil-Cre*^+^ DRG neurons compared to controls (Supplementary Figure 4A). The difference was not observed between the TAM-injected *Prdm12*^*fl*/*fl*^; *Rosa26-CreER*^*T*2+^ and littermate controls. To determine the contribution of tetrodotoxin- (TTX-) sensitive and resistant inward currents observed in *Prdm12*^*fl*/*fl*^*; Avil-Cre*^+^, subsequent inhibition of TTX-sensitive inward currents was performed by 250 ms prepulse at −40 mV showing a reduction of inward currents only in *Prdm12*^*fl*/*fl*^; *Avil-Cre*^+^, and not in TAM-injected *Prdm12*^*fl*/*fl*^; *Rosa26-CreER*^*T*2+^ neurons (Supplementary Figure 4B), as compared to their respective littermate controls. These results imply a larger fraction of TTX-S Na+ channels in *Prdm12*^*fl*/*fl*^*; Avil-Cre*^+^ (Supplementary Figures 4C–E).

PR domain-containing member 12-deficient and control neurons were also tested for their thermal responsiveness by an increase in a ramp-shaped temperature. Strikingly, only a single *Prdm12*^*fl*/*fl*;^*Avil-Cre*^+^ neuron (of the 42 recorded neurons) responded to the thermal stimulus with a relatively high temperature threshold (>45.0°C, Figure 4G). From the *Prdm12*^*fl*/*fl*^*; Avil-Cre*,, ~38% of the neurons responded with an averaged threshold temperature of 40.0°C. The percentage of thermal-responsive neurons was similar between TAM-injected *Prdm12*^*fl*/*fl*^; *Rosa26-CreER*^*T*2+^ and *Prdm12*^*fl*/*fl*^; *Rosa26-CreER*^*T*2−^ neurons and the temperature threshold of the thermal-activated currents (Figures 4H,I).

To determine the behavioral responses of our mouse models to noxious chemical stimuli, we injected intraplantar capsaicin, a pungent ingredient in chili peppers, and recorded the behavioral response. Capsaicin activates its receptor TrpV1 on nociceptive C-fibers, generating a painful sensation completely absent in CIP patients. We found that both conditional mouse models *Prdm12*^*fl*/*fl*^*; Avil-Cre*^+^ and TAM-injected *Prdm12*^*fl*/*fl*^; *Rosa26-CreER*^*T*2+^ had significantly reduced responses to capsaicin as compared to their respective control littermates (Figures 4H,J).

### Developmental or Adult-Onset Deletion of PRDM12 Causes Different Effects in Downstream Gene Expression

**Figure 5:**
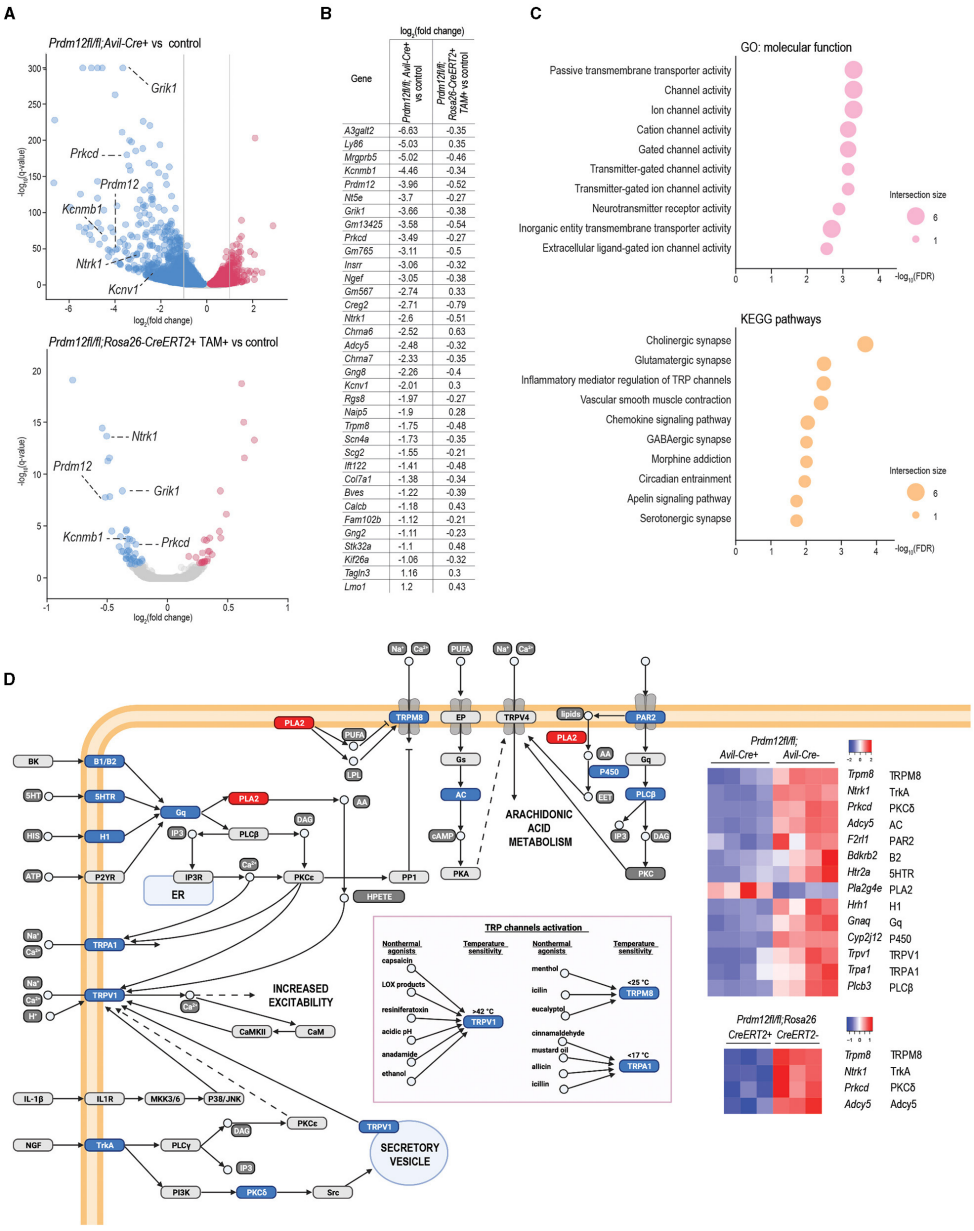
Transcriptomics analysis of DRG of Prdm12-deficient animal models. **(A)** Volcano plot of differentially expressed genes (DEGs) depicts significantly upregulated and downregulated genes in pooled lumbar DRG isolated from adult *Prdm12*^*fl*/*fl*^*; Avil-Cre*^+^ (upper panel, four biological replicates) and *Prdm12*^*fl*/*fl*^*; Rosa26-ER*^*T*2+^ (lower panel, three biological replicates) as compared to their respective control littermates. X-axis represents log_2_ fold change and the y-axis represents –log_10_(*q*-values). Genes with the value of *q* (FDR) < 0.05 were considered differentially regulated, downregulated genes in blue and upregulated genes in red. Gray dotted lines in the top panel demark the cutoff for the most DEGs with at least 2-fold upregulation or downregulation. **(B)** The list of core DEGs (value of *q* < 0.05) common to both *Prdm12*^*fl*/*fl*^*; Rosa26-ER*^*T*2+^ and *Prdm12*^*fl*/*fl*^*; Avil-Cre*^+^ model, and the most DEGs (value of *q* < 0.05, |log2(fold change)|>1) of the *Prdm12*^*fl*/*fl*^*; Avil-Cre*^+^ model as compared to their corresponding controls. **(C)** About 10 most enriched terms in the overrepresentation analysis of the core DEGs for the GO:MF database (upper panel) and kyoto encyclopedia of genes and genomes (KEGG) pathways (lower panel). Values on the x-axis present –log10 of the Benjamini–Hochenberg FDR score for a particular pathway enrichment significance. **(D)** Transient receptor potential (TRP) channels pathway with heatplot of *Z*-scores for DEG members of the TRP pathway in both developmental and adult Prdm-12 deficiency model. Protein symbols corresponding to the dysregulated transcripts are denoted next to the transcript symbol, and colors within the pathway illustration correspond to the downregulation (blue) or upregulation (red) of the transcripts represented in the heatmap.

To identify the effect of *Prdm12* deletion on the key genes responsible for acute pain perception between our two mouse models, we performed mRNA sequencing on bulk lumbar DRG samples from at least three animals of both models and corresponding controls. We sought to clarify the cellular phenotypic difference of the development and adult mouse model. *Prdm12*^*fl*/*fl*^*; Avil-Cre*^+^ had 5,851 significantly (false discovery rate, FDR < 0.05) DEGs as compared to corresponding controls, including markers of nociceptors, some of which including *Trpv1, Ntrk1, Calca*, and *Scn10a* are additionally validated by RT-qPCR (Supplementary Figure 5A). Out of 5,851 DEGs, 868 genes had an expression level fold change larger than 2 for upregulated DEGs, or smaller than −2 for downregulated DEGs (Figure 5A, top panel). In comparison, TAM-injected *Prdm12*^*fl*/*fl*^; *Rosa26-CreER*^*T*2+^ had 67 significantly (FDR < 0.05) DEGs (Figure 5A, bottom panel).

To gain insights into the possible contribution of DEGs to the PRDM12 core transcriptional program crucial for the function of nociceptors, we considered all 67 *Prdm12*^*fl*/*fl*^; *Rosa26-CreER*^*T*2+^ DEGs and 868 most significantly dysregulated genes of the *Prdm12*^*fl*/*fl*^*; Avil-Cre*^+^ model and identified 35 transcripts common to both models, now referred to as PRDM12 core DEGs (Figure 5B). In addition to *Prdm12*, the core DEGs include the genes encoding for TRP channels and TRP channel-associated signaling including *Ntrk1, Trpm8, Adcy5, Prkcd*, and ion channels like *Kcnv1, Kcnb1, Scn4a*, and neurotransmitter receptor activity like *Grik1* and *Chrna7*. Overrepresentation analysis of MF and KEGG pathway gene sets with a g:profiler toolbox revealed that PRDM12 core DEGs are mainly involved in the function of ion channel activity, either passive or gated (Figure 5C). Among KEGG pathways, top enriched pathways are associated with synaptic signaling and pathways regulating TRP-mediated signaling (Figure 5C, bottom panel). DEG protein products included in TRP channel signaling are illustrated in Figure 5D, with the associated heat map representing DEGs from both mouse models.

**Figure 6:**
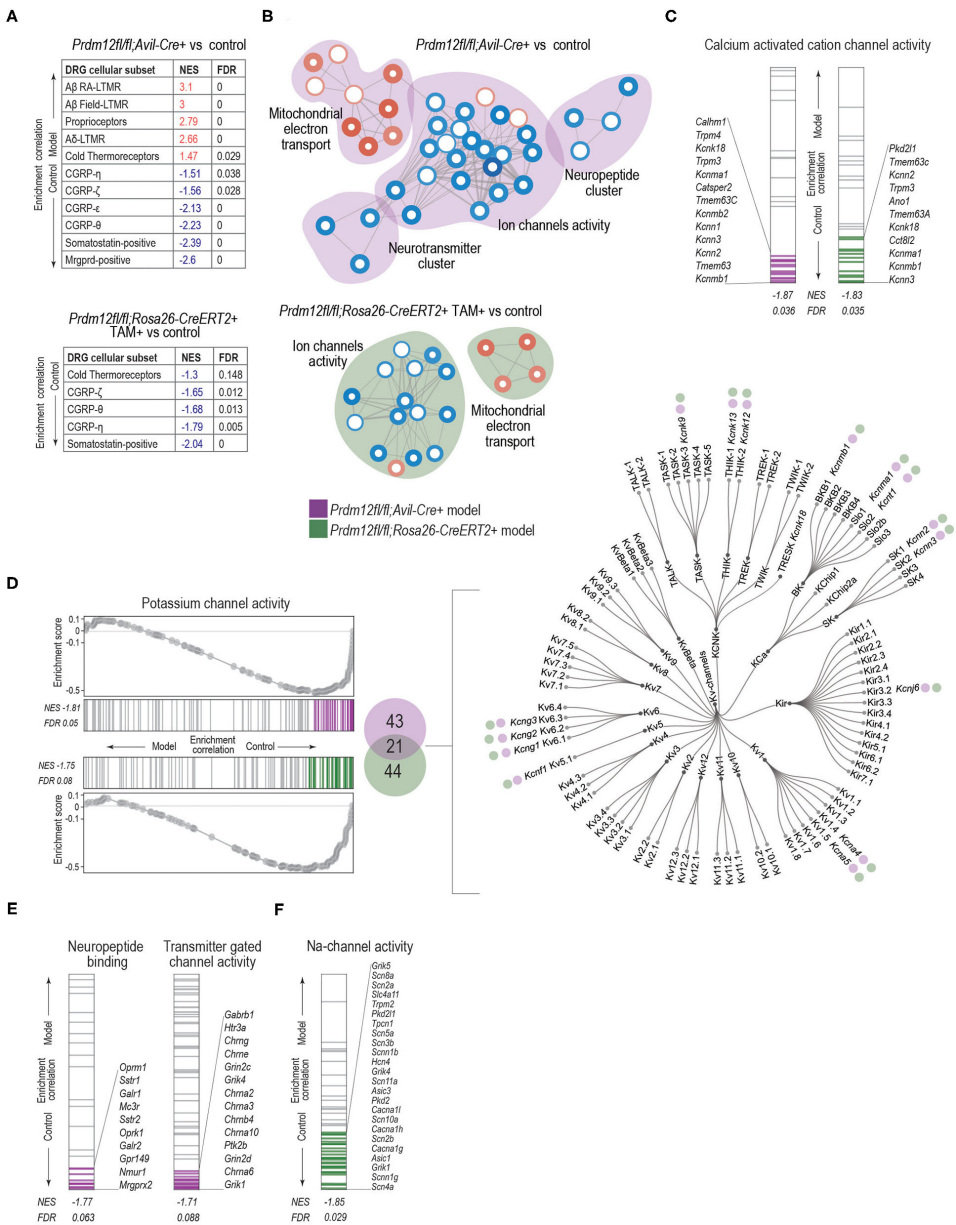
GSEA analysis with enrichment mapping shows distinct clusters of dysregulated MF:GO terms. **(A)** Dysregulation of bulk RNA DRG transcriptomes as demarked by gene sets most significant by sensory neuron subtypes detected in the DRG, for both *Prdm12*^*fl*/*fl*^*; Avil-Cre*^+^ (top table) or TAM-injected *Prdm12*^*fl*/*fl*^*; Rosa26-ER*^*T*2+^ (bottom table) as detected by GSEA with the cutoff value of *p* of 0.05 and FDR cutoff of 0.25. **(B)** Clusters of GSEA enriched-term nodes in the GO:MF terms. Nodes in red either in *Prdm12*^*fl*/*fl*^*; Avil-Cre*^+^ (pink cloud, left) or in *Prdm12*^*fl*/*fl*^*; Rosa26-ER*^*T*2+^ (blue cloud, right) are enriched in DRG, while nodes in blue represent the depletion of MF terms, as labeled. Thickness of the node border corresponds to the –log_10_(FDR), while depth of the color corresponds to normalized enrichment score (NES) of a particular node as compared to the whole gene set. Thickness of the edge lines corresponds to the strength of the used similarity coefficient. Clusters of highly connected nodes are grouped together and annotated with the closest common functional term. **(C)** GSEA enrichment graphs showing the distribution of calcium-activated cation channel activity as an example of a significantly enriched GO:MF term common to both in *Prdm12*^*fl*/*fl*^*; Avil-Cre*^+^ (left) or TAM-injected *Prdm12*^*fl*/*fl*^*; Rosa26-ER*^*T*2+^ (right) showing genes contributing to the leading edge in purple and green for *Prdm12*^*fl*/*fl*^*; Avil-Cre*^+^ and *Prdm12*^*fl*/*fl*^*; Rosa26-ER*^*T*2+^, respectively. NES and FDR values are denotated in the figure. **(D)** Enrichment graphs depicting the distribution of GOM:MF potassium channel activity genes enrichment in the controls of both *Prdm12*^*fl*/*fl*^*; Avil-Cre*^+^ (left panel, top) and *Prdm12*^*fl*/*fl*^*; Rosa26-ER*^*T*2+^ (left panel, bottom). Graphs for each model show running enrichment scores, as well as NES and FDR. Genes within the leading edge are colored purple and green for *Prdm12*^*fl*/*fl*^*; Avil-Cre*^+^ and *Prdm12*^*fl*/*fl*^*; Rosa26-ER*^*T*2+^ models, respectively. In the middle panel, Venn diagram depicts the number of genes within the leading edge of *Prdm12*^*fl*/*fl*^*; Avil-Cre*^+^ (43 genes, purple) and *Prdm12*^*fl*/*fl*^*; Rosa26-ER*^*T*2+^ (44 genes, green) and 21 overlapping genes. Dendogram of voltage-gated potassium channel families (right) denotes the type of Kv-channel transcripts shared between the two models, note the purple and green circles. **(E)** Enrichment graphs showing the distribution of terms specific for *Prdm12*^*fl*/*fl*^*; Avil-Cre*^+^ model, showing the depletion of GO:MF neuropeptide binding genes and transmitter-gated channel activity genes in *Prdm12*^*fl*/*fl*^*; Avil-Cre*^+^ model as compared to the corresponding controls. Positions of genes contributing to the significant enrichment are colored in purple. **(F)** Enrichment graphs showing the distribution of GO:MF sodium channel activity genes in the transcriptome of *Prdm12*^*fl*/*fl*^*; Rosa26-ER*^*T*2+^ model, showing an enrichment in the controls as compared to the deficiency model. Positions of genes contributing to the significant enrichment are colored in green. NES and FDR values are denoted in the figures.

To examine if dysregulated transcriptomes of *Prdm12*^*fl*/*fl*^*; Avil-Cre*^+^ and TAM-injected *Prdm12*^*fl*/*fl*^; *Rosa26-CreER*^*T*2+^ models vs. corresponding controls reflect transcriptome footprints of nociceptors or some other sensory cell types in the DRG as benchmark gene sets, we used the top 50 genes marking each of the 14 different distinct sensory cellular subtypes described by Sharma et al. (2020) and performed GSEA. *Prdm12*^*fl*/*fl*^*; Avil-Cre*^+^ model is predominantly enriched for the cell types associated with proprioception and mechanoreception (Figure 6A, top). Moreover, in both *Prdm12*^*fl*/*fl*^*; Avil-Cre*^+^ and *Prdm12*^*fl*/*fl*^; *Rosa26-CreER*^*T*2+^ experimental set-up, the subtypes of nociceptors were enriched in the corresponding controls as compared to the loss-of-function models (Figure 6A, Supplementary Figures 5B–D). This finding shows that the deletion of *Prdm12* depletes DRG of nociceptor-specific genes, and in particular the subtypes of somatostatin-positive and CGRP-positive nociceptors. Intriguingly, this observation is common to both the developmental *Prdm12*^*fl*/*fl*^*; Avil-Cre*^+^ as well as adult TAM-inducible *Prdm12*^*fl*/*fl*^; *Rosa26-CreER*^*T*2+^models. Recently, Landy et al. (2021) have reported a transcriptomic analysis of bulk RNA in DRG harvested from a spatially restricted adult-inducible Prdm12 KO (*Prdm12Avil*^*ERT*2*CKO*^). Between 150 genes that were dysregulated in TAM-inducible *Prdm12Avil*^*ERT*2*CKO*^ described by Landy et al. (2021) and 67 DEGs that were found in our TAM-inducible *Prdm12*^*fl*/*fl*^; *Rosa26-CreER*^*T*2^ model, we find an overlap in 2 genes—*Prdm12* and *Chrna6*. However, between DEGs in Landy et al. (2021) model and our *Prdm12*^*fl*/*fl*^*; Avil-Cre* model, we identified 40 overlapping genes, 21 of which are expressed in the DRG, including *Prdm12, Chrna6, Trpm3*, and are important for the development of nervous system, such as *Nnat*. Mapping of the possible functional consequences of the DEGs in both models was performed by GSEA of GO MF (GO:MF) gene sets, followed by generating the clustered network of the enriched terms (Figure 6B). Developmental model *Prdm12*^*fl*/*fl*^*; Avil-Cre*^+^ vs. controls exhibited an enrichment in the control samples (conversely the depletion in *Prdm12*^*fl*/*fl*^*; Avil-Cre*^+^) for gene sets that cluster into networks including “ion channel activity,” “neurotransmitter associated activity,” and “neuropeptides” (Figure 6B). Clusters in the adult model TAM-injected *Prdm12*^*fl*/*fl*^; *Rosa26-CreER*^*T*2+^ vs. control maintain the depletion of gene sets associated with “ion channel activity” (Figure 6A).

To explore the enrichment patterns within ion channel activity clusters between the models, we investigated various gene sets to identify if any specific GO:MF terms are common in the two models. “Calcium activated ion channel activity” (Figure 6C) and “potassium channel activity” terms (Figure 6D, Supplementary Figure 5E) are enriched in controls of both models. Within the genes contributing to the leading edge of both models, 21 genes are common (Figure 6D, middle panel). Most of the common genes belong to the group of voltage-gated potassium channels (Figure 6D, right panel). To identify specific gene sets that differ between *Prdm12*^*fl*/*fl*^*; Avil-Cre*^+^ and *Prdm12*^*fl*/*fl*^; *Rosa26-CreER*^*T*2+^ models, we analyzed neuropeptide and neurotransmitter clusters in the *Prdm12*^*fl*/*fl*^*; Avil-Cre*^+^. Within a neuropeptide cluster, GO terms associated with neuropeptide binding were depleted in the *Prdm12*^*fl*/*fl*^*; Avil-Cre*^+^ (and enriched in the controls), with transcripts associated with opioid receptors *Oprm1* and *Oprk*, as well as galanin receptors *Galr1* and *Galr2* within the leading edge (Figure 6E, left panel). In the neurotransmitter cluster, genes associated with neurotransmitter-mediated ion channel activity were depleted in *Prdm12*^*fl*/*fl*^*; Avil-Cre*^+^ model as compared to controls, with the subunits of glutamate receptors (*Grin2c, Grik4, Grin2d*, and *Grik1*), cholinergic nicotinic receptors contributing the most to the leading edge (Figure 6E, right panel). Furthermore, the analysis of ion channel cluster in the *Prdm12*^*fl*/*fl*^; *Rosa26-CreER*^*T*2+^ model revealed the depletion of sodium channel activity genes, including markers of nociceptors, voltage-gated sodium channels encoded by *Scn11a*, and *Scn10a*, members of TRP pathways like acid sensing channels encoded by *Asic1* and *Asic3* as well as glutamate receptor subunits *Grik1, Grik4*, and *Grik5* (Figure 6F).

## Discussion

Here, we present two novel cases of CIP in a large, consanguineous family, exhibiting symptoms in line with defective nociception that share a novel homozygous variant in *PRDM12*. Generating several developmental and adult-onset deletion mouse models of *Prdm12*, we could determine that early developmental expression of PRDM12 is essential for survival in mice. We show that PRDM12 expression is essential for acute noxious pain detection throughout life. Detailed electrophysiological analyses revealed different but significantly altered biophysical properties of cultured DRG in the developmental and in the adult PRDM12 ablation models. Indeed, different transcriptional programs are regulated by PRDM12 at different maturation stages, accounting for a difference in the corresponding cellular phenotypes. Taken together, these data confirm that developmental PRDM12 is essential in pain perception in mice by regulating key nociceptor-specific genes required for the development and function of nociceptors. While PRDM12 expression in adults is no longer essential for survival or needed for certain types of CNS functions, it is still required for nociceptive responses.

Human PRDM12 expression is exclusive to the PNS making it a promising therapeutic target (Chen et al., 2015). However, considering the restricted PRDM12 expression, it is not clear why a small number of PRDM12-CIP patients, including those in this report, have CNS dysfunctions. The analysis of WES of patient 1 identified eight rare variants in genes associated with autism and/or ID, but these are not shared with patient 2 and are unlikely to explain the CNS symptoms in this family. Interestingly, a splice-site acceptor variant reported here is in the same position as an already reported PRDM12-CIP case of different ethnic background, presenting with typical CIP symptoms including ID (Saini et al., 2017). Why should variants at this position but not in others have an impact on CNS function is not clear. Therefore, the molecular mechanisms underlying CNS symptoms that are associated with PRDM12-CIP in a small number of cases remain to be elucidated.

We report PRDM12 expression in all adult mouse brain regions examined, suggesting a role for PRDM12 beyond nociception. Generating the *Prdm12*^*W*160*C*^ mouse model that proved to be neonatally lethal allowed us to conclude that fully functional PRDM12 is required for the survival in mice. While the cause of death is still not clear, lethality of the neuronal progenitor KO model *Prdm12*^*fl*/*fl*^; *Sox10-Cre* leads us to posit that the PRDM12 essentiality may be intrinsic to the cells derived from the Sox10+ lineage, not necessarily of neuronal origin. The heart or brain, for example, expresses PRDM12 but also contain Sox10+ cardiomyocytes and glia, respectively (Kuhlbrodt et al., 1998; Montero et al., 2002; Sande-Melón et al., 2019; Tang et al., 2019). The role for PRDM12 in mouse CNS has recently gained attention. Hypothalamic PRDM12 regulates *Pomc* expression, food intake, adiposity, and weight (Chen et al., 2020; Woo et al., 2020). Here, we report no effect of ablating PRDM12 expression in adults on survival, locomotion, anxiety, or hippocampal-dependent learning. These results suggest that whatever the role for developmental PRDM12 is in the mouse CNS, it is no longer required for the behaviors tested here in the adult. It would be of great interest to fully characterize the role for PRDM12 in the mouse CNS, and in light of a growing number of PRDM12-CIP patients with CNS symptoms, to explore the possibility for its function in the human brain.

Analyses of PRDM12 developmental and adult-ablation models show a dependence of proper nociceptive responses on the presence of Prdm12. Contrary to our results, recent publication indicates that, while the adult murine model of Prdm12 deficiency has an effect on transcriptional regulation, it does not translate to the impairment of pain sensation behaviorally (Landy et al., 2021). We posit that these differences could be explained by the different experimental mouse models used in the two studies. TAM-inducible *Prdm12*^*fl*/*fl*^; *Rosa26-CreER*^*T*2+^ adult model used here targets *Prdm12* exon 2, mimicking the splice acceptor mutation described in our patients, under the ubiquitous *Rosa26* promoter, while Landy et al. (2021) describe a model that targets exon 5, and is under the TAM-inducible DRG-specific *Avil* promoter targeting a more specific cellular subtype only in the periphery. The reduction in behavioral responses to capsaicin injections together with the significant dysfunction of cultured nociceptor electrophysiological responses in our *Prdm12*^*fl*/*fl*^; *Rosa26-CreER*^*T*2+^ mouse model confirm PRDM12 nociceptive regulation in the PNS even in adults. However, a variety of behavioral pain responses were not observed in the DRG-specific adult-ablation model in Landy et al. (2021), implying a possible contribution of PRDM12 action in CNS pain responses that would warrant further study.

Electrophysiological studies of developmental homozygous mouse model clearly determined that it is the peripherally expressed PRDM12 during the embryonic/developmental stage that is required for the detection and signaling of nociceptive stimuli. We find that, while resting membrane potential is unaffected in the developmental PRDM12-ablation model, AP morphology is impaired, as well as the number of APs generated upon different stimulation protocols. Moreover, based on the prepulse inhibition experiments, we find that the ratio of currents generated by TTX-sensitive Na^+^ channels is increased in *Prdm12*^*fl*/*fl*^*; Avil-Cre*^+^ neurons as compared to control littermates. A significant reduction of pain-related genes encoding for Na_v_1.8, Na_v_1.9, TrpV1, TrkA, and CGRP, in the *Prdm12*^*fl*/*fl*^*; Avil-Cre*^+^ strongly corroborates the electrophysiological nociceptive dysfunctions are due to the lack of nociceptors in developmental models as described previously (Desiderio et al., 2019).

Transcriptome analysis of the bulk DRG shows *Prdm12*^*fl*/*fl*^*; Avil-Cre*^+^ dysregulation of more than 5,000 genes, 868 of which are dysregulated by at least a 2-fold increase or decrease. Downregulated expression of nociceptor signature genes including *Ntrk1, Scn10a* and *Scn11a, Calca* in *Prdm12*^*fl*/*fl*^*; Avil-Cre*^+^ compared to controls is a consequence of the lack of nociceptor development as described previously (Desiderio et al., 2019), and confirmed by the lack of skin PGP9.5 positive innervation in *Prdm12*^*fl*/*fl*^*; Avil-Cre*^+^ animals. Nevertheless, as *Prdm12*^*fl*/*fl*^; *Rosa26-CreER*^*T*2+^ TAM-inducible model overlaps with *Prdm12*^*fl*/*fl*^*; Avil-Cre*^+^ in 35 DEGs, which in the overrepresentation analysis is associated with ion channels, and more specifically TRP channels pathways, indicate that Prdm12 maintains a role in regulating ion channels essential for the function of nociceptors even in the adulthood, supported by the electrophysiological and behavioral data. Indeed, the comparison of our *Prdm12*^*fl*/*fl*^*; Avil-Cre* model transcriptomic data to adult TAM-inducible model *Prdm12Avil*^*ERT*2*CKO*^ proposed by Landy et al. (2021) shows an overlap in 40 DEGs, out of which 21 are DRG genes.

Gene set enrichment analysis highlights that both developmental and adult models share the depletion of the terms associated with an ion channel cluster. Many of these terms in adult model are the members of distinct subfamilies of voltage-gated potassium channels essential for the generation and propagation of APs, corroborating the electrophysiological changes and behavioral responses to capsaicin injections. In contrast, developmental model exhibits specific clusters of dysregulated gene sets including neurotransmitter- (e.g., *Grik1, Grik4, Grin2d*, and *Grin2c*) and neuropeptide- (e.g., *Oprm1, Oprk1, Galr1, Galr2*, and *Nmur1)* related clusters. Specific gene set that is selectively depleted in *Prdm12*^*fl*/*fl*^; *Rosa26-CreER*^*T*2+^ but not in *Prdm12*^*fl*/*fl*^; *Avil-Cre*^+^ is associated with sodium channel activity. Interestingly, these include nociceptor-specific genes *Scn10a*, encoding for Na_v_1.8 and *Scn11a* encoding for Na_v_1.9, possibly contributing to the observed differences in AP firing in TAM-injected *Prdm12*^*fl*/*fl*^; *Rosa26-CreER*^*T*2+^ as compared to the corresponding controls.

*PR domain-containing member 12* ablation in *Prdm12*^*fl*/*fl*^*; Avil-Cre*^+^ animals results in electrophysiological and behavioral deficiencies and the lack of nociceptor skin innervation due to the essential role played by PRDM12 in the specification of nociceptor development. However, TAM-injected *Prdm12*^*fl*/*fl*^; *Rosa26-CreER*^*T*2+^ adult-deletion model maintains a significant dysregulation of a small subset of genes involved in TRP channel and ion channel activities. Clearly, PRDM12 is an indispensable component of nociceptor specification and function required for pain perception throughout life. Together with its uniquely restricted expression in humans and the requirement for adult pain perception make it an intriguing therapeutic target with immense clinical potential.

## Data Availability Statement

The datasets presented in this study can be found in online repositories. The names of the repository/repositories and accession number(s) can be found at: https://www.ncbi.nlm.nih.gov/, PRJNA734482.

## Ethics Statement

The studies involving human participants were reviewed and approved by Shahid Beheshti University of Medical Sciences ethics board (with the current update of the Declaration of Helsinki). Written informed consent to participate in this study and for the publication of any potentially identifiable images or data included in this article was provided by the participants' legal guardian/next of kin. The animal study was reviewed and approved by Bundesministerium fur Wissenschaft, Forschung und Wirtschaft (BMWFW-66.015/0011-WF/V/3b/2017), Austria and carried out according to EU-directive 2010/63/EU. Prdm12W160 and Prdm12S159AfsTer2+/- experiments were approved by the University of Sydney Animal Ethics Committee under animal ethics protocol (938) and reporting complies with the ARRIVE guidelines.

## Author Contributions

TK: curated data, performed the formal analysis, investigation, validation of the data, visualization, and assisted in writing the manuscript. ML: curated data, performed the formal analysis, investigation, and assisted in writing the manuscript. EL, JM, and CF: curated data, performed the formal analysis, and investigation. EA and AT: curated patient data. HD: participated in project administration and supervision. EB: provided resources. GN and MK: provided resources, project administration, supervision, and funding acquisition. JP: conceptualized the project and methodology, provided resources, and funding acquisition. VN: conceptualized the project and methodology, curated data, participated in the investigation, formal analysis, resources, project administration, supervision, funding acquisition, and wrote the manuscript. All authors contributed to the article and approved the submitted version.

## Funding

This work was funded by Austrian Science Fund (FWF): P32924 and Ludwig Boltzmann Gesellschaft. TK was funded by a DOC-fellowship of the Austrian Academy of Sciences (OeAW): 25408; CF was funded by a DOC-fellowship of the Austrian Academy of Sciences (OeAW): 25525.

## Conflict of Interest

The authors declare that the research was conducted in the absence of any commercial or financial relationships that could be construed as a potential conflict of interest.

## Publisher's Note

All claims expressed in this article are solely those of the authors and do not necessarily represent those of their affiliated organizations, or those of the publisher, the editors and the reviewers. Any product that may be evaluated in this article, or claim that may be made by its manufacturer, is not guaranteed or endorsed by the publisher.
