# Supplementary material for: PRDM12 Is Transcriptionally Active and Required for Nociceptor Function Throughout Life
Source: Front Mol Neurosci. 2021 Sep 27;14:720973. doi: 10.3389/fnmol.2021.720973 (PMC8502974; doi:10.3389/fnmol.2021.720973)
Supplement: Supplementary file 1 [file Data_Sheet_1.docx]

**Supplemental Information**

**Supplemental Materials and Methods**

**Single cell electrophysiology**

In voltage clamp recordings, the neurons were clamped at a holding potential of -60mV. Cells were challenged with twice with IV-protocol from -80mV to +40mV (50ms, 5mV steps), one IV-protocol was preceded by a 250ms prepulse of -120mV (IV_PP-120_) and the other preceded by a 250ms -40mV prepulse (IV_PP-40_) to assess the amount of TTX-resistant inward currents. Currents were recorded at 20kHz. Each IV was preceded by an automated cellular capacitance compensation. A leakage subtraction protocol (P/N 5) was applied preceding all current recordings all currents were normalized for the capacitance of the recorded cell (current density in pA.pF^-1^).

*Analysis of voltage-gated inward currents*

Peak inward currents were plotted against voltage and were fitted with a modified Boltzmann equation with one-step activation phase

$$I= I_{Leak}+G*\left( \frac{1}{1+e \left( \frac{V-V_{act}}{S_{act}} \right)} \right)*\left( V-V_{rev} \right)$$

where includes conductance G (nS.pF^-1^), Slope S (mV), half-activation voltage V_act_ (mV) and the reversal potential (V_rev_ in mV).

**Supplemental Figure Legends**

**Figure S1**. **Genotype confirmation and behavioral nociceptive phenotyping of *Prdm12^S159AfsTer2+/-^* mice. A)** Sanger chromatogram confirming the c.537A_del frameshift (left panel), WT for comparison (right panel) generated by CRISPR-Cas9. **B)** Profiling of heterozygous *Prdm12^S159AfsTer2+/-^* (N=16) in von Frey (touch, two left panels) and Hargreaves (heat, two right panels) at both low (IR 40) and high (IR 70) intensity tests indicated no overt somatosensory phenotype as compared to littermate controls (N=18). IR-irradiation. Mean values ± SEM.

**Figure S2. Confirmation of *Prdm12* downregulation and survival of the *Prdm12^fl/fl^;Avil-Cre^+^* model animals. A)** RT-qPCRs (left) of adult lumbar DRG tissue of *Prdm12^fl/fl^;Avil-Cre^+^* show a near complete reduction of *Prdm12* transcript as compared to control littermates, supported by protein expression reduction of PRDM12 in the DRG tissue (Western blot, right panel). **B)** 5 breeder cages with 2 female *Prdm12^fl/fl^;Avil-Cre^-^* and 1 male *Prdm12^fl/fl^;Avil-Cre^+^* were scored for a number of offspring that reached weaning age for a given amount of time. Total of 94 animals were counted. Expected ratio of offspring genotype was 50% *Prdm12^fl/fl^;Avil-Cre^+^* and 50% *Prdm12^fl/fl^;Avil-Cre^-^*. Pie chart represents the actual ratio of surviving pups. **C)** Image of typical facial and eye scarring of adult *Prdm12^fl/fl^;Avil-Cre^+^* mouse. P-values * ≤ 0.05, ** ≤ 0.01, *** ≤0.005, ****≤ 0.0001.

**Figure S3. Open Field and Morris Water Maze behavioral assays of the *Prdm12^fl/fl^;Rosa26-CreER^T2^* animals. A)** RT-qPCRs of adult TAM-injected *Prdm12^fl/fl^;Rosa26-CreER^T2+^* lumbar DRG tissue (left panel) and whole brain tissue (right panel) show significant reduction of *Prdm12* transcript as compared to control littermates. **B)** Open Field Test determined that *Prdm12^fl/fl^;Rosa26-CreER^T2+^* (N=8) spend similar amount of time along the border (left panel) and in the center (middle panel) of the arena as compared to control littermates (N=12). TAM-injected *Prdm12^fl/fl^;Rosa26-CreER^T2+^* also traveled similar distance in the arena as their littermate controls (130m vs. 117.4m, right panel). **C)** Morris Water Maze shows no difference in decreasing latency over the 5 trial days to find the hidden platform between the genotypes (acquisition, upper panel). Both genotypes spent similar amount of time in the target quadrant in the short-term probe trial (40.15% vs. 45.1%, lower left panel) and long-term probe trial (43.0% vs. 39.8% lower right panel). Mean values ± SEM. P-values * ≤ 0.05, **≤ 0.01, ***≤ 0.005, ****≤ 0.0001.

**Figure S4. Characterization of inward currents in PRDM12 ablated sensory neurons. A)** Current-voltage (IV), preceded by a 250ms prepulse at -120mV (IVpp-120), showed that *Prdm12^fl/fl^;Avil-Cre^+^* have increased inward currents compared to its respective *Prdm12^fl/fl^;Avil-Cre^-^* controls. Ablation of *Prdm12* in the adult stage, *Prdm12^fl/fl^;Rosa26-CreER^T2+^* did not increase the inward currents. **B)** Inhibition of TTX-sensitive inward currents by a 250ms prepulse at -40mV (IVpp-40) shows a reduction of inward currents in *Prdm12^fl/fl^;Avil-Cre^+^* sensory neurons. Again no differences are observed between the *Prdm12^fl/fl^;Rosa26-CreER^T2+^* and *Prdm12^fl/fl^;Rosa26-CreER^T2-^* sensory neurons. **C)** In accordance with the increased inward currents of *Prdm12^fl/fl^;Avil-Cre^+^* derived from IVpp-120, the inward conductance (G) is significantly larger compared to *Prdm12^fl/fl^;Avil-Cre^-^*, whereas the activation voltage (V_act_) and slope of activation (S) are not changed. **D)** Analysis of the IVpp-40 plots did not show any differences in the inward conductance between the PRDM12-ablated sensory neurons and their controls. However, in V_act_ and slope of *Prdm12^fl/fl^;Avil-Cre^+^* recorded currents are significantly decreased and increased respectively. No differences are observed in the *Prdm12^fl/fl^;Rosa26-CreER^T2+^* DRG neurons. **E)** In *Prdm12^fl/fl^;Avil-Cre^+^*, the balance of summed (IVpp-120-IVpp-40) and IVpp-40 inward currents is significantly changed compared to *Prdm12^fl/fl^;Avil-Cre^+^*, whereas ablation of *Prdm12* in *Prdm12^fl/fl^;Rosa26-CreER^T2+^* DRG neurons did not cause a shift in the balance. P-values * ≤ 0.05, **≤ 0.01, ***≤ 0.005, ****≤ 0.0001.

**Figure S5. Distinct nociceptor cellular subtypes are dysregulated in developmental and adult models. A)** RT-qPCR validation of bulk RNA-seq for the expression levels of selected markers of nociceptor identity in *Prdm12^fl/fl^;Avil-Cre^+^* DRG as compared to the corresponding controls. **B-D)** Gene set enrichment analysis plots for using gene sets specific for 14 different sensory neuronal subtypes and applied to both *Prdm12^fl/fl^;Avil-Cre^+^* and TAM-injected *Prdm12^fl/fl^;Rosa26-CreER^T2+^* DRG bulk RNA-seq as compared to corresponding controls. Enrichment plots show **B)** cellular subtypes enriched *Prdm12^fl/fl^;Avil-Cre^+^* depletion model **C)** cellular subtypes depleted *Prdm12^fl/fl^;Avil-Cre^+^* depletion model **D)** cellular subtypes depleted *Prdm12^fl/fl^;Rosa26-CreER^T2+^* depletion model. NES and FDR values are showed under each plot. Used gene sets available in supplementary table as .gmt file. **E)** Dfferentially expressed voltage gated potassium channel genes in *Prdm12^fl/fl^;Avil-Cre^+^* model. Volcano plot of differentially regulated genes depicts significantly up- and down-regulated genes in pooled DRG isolated from adult *Prdm12^fl/fl^;Avil-Cre^+^* X-axis represents log_2_ fold change and the y-axis represents –log_10_(q-values). Genes with q-value (FDR) of less than 0.05 (marked with horizontal line) were assigned differentially regulated and colored in blue if downregulated or in red if upregulated. Additional lines perpendicular to y-axis in the top panel demark the cutoff for the most dysregulated genes with at least 2 fold up- or downregulation. Positions of differentially regulated genes for voltage gated potassium channels are labeled with purple perimeter. DRG samples from 4 biological replicates for *Prdm12^fl/fl^;Avil-Cre^+^* and control were analyzed, and 3 biological replicates for *Prdm12^fl/fl^;Rosa26-ER^T2+^* were used and analyzed.
