# Supplementary figures and images for: PRDM12 Is Transcriptionally Active and Required for Nociceptor Function Throughout Life
Source: Front Mol Neurosci. 2021 Sep 27;14:720973. doi: 10.3389/fnmol.2021.720973 (PMC8502974; doi:10.3389/fnmol.2021.720973)

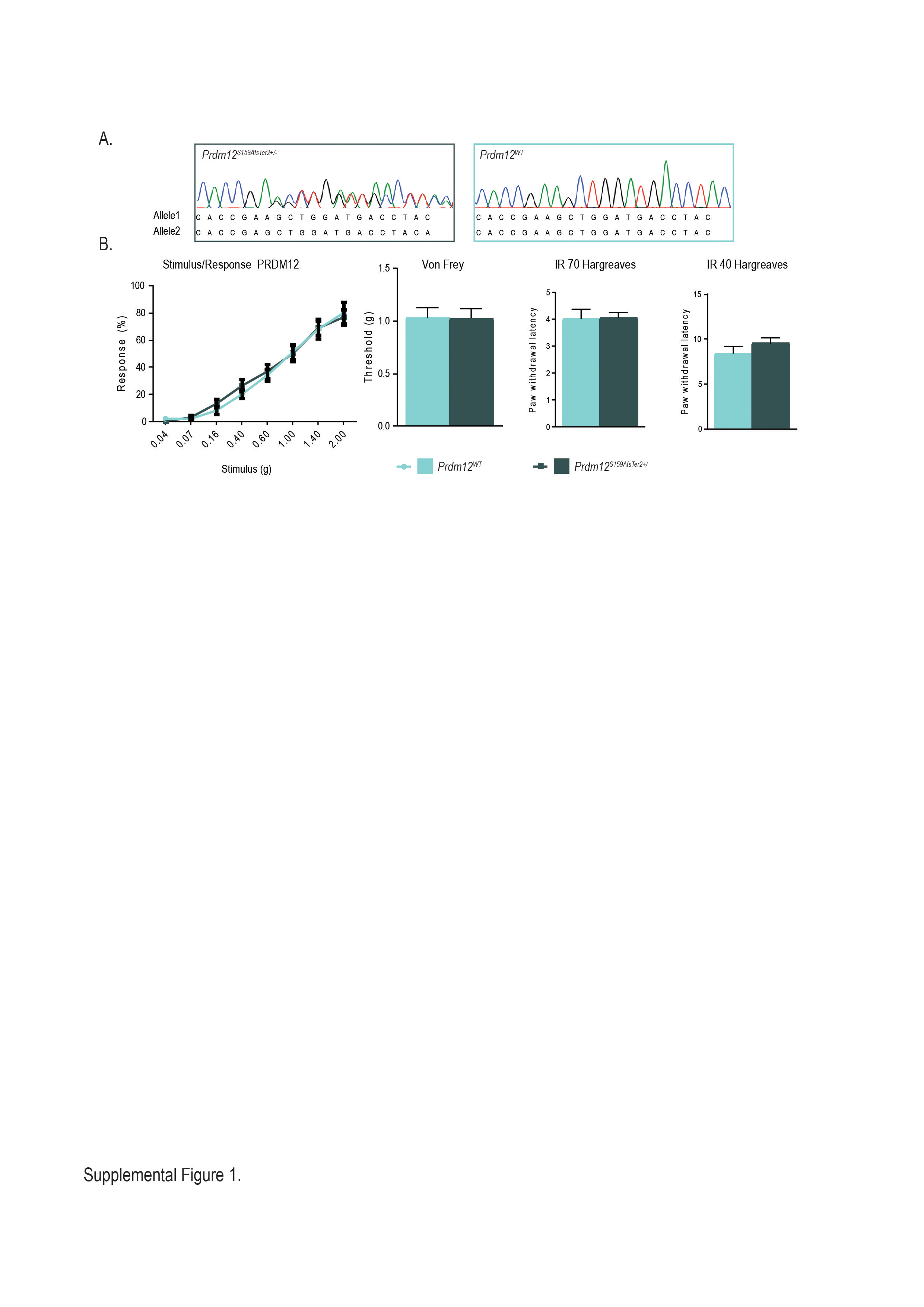

Supplement: Supplementary file 3 [file Image_1.JPEG]

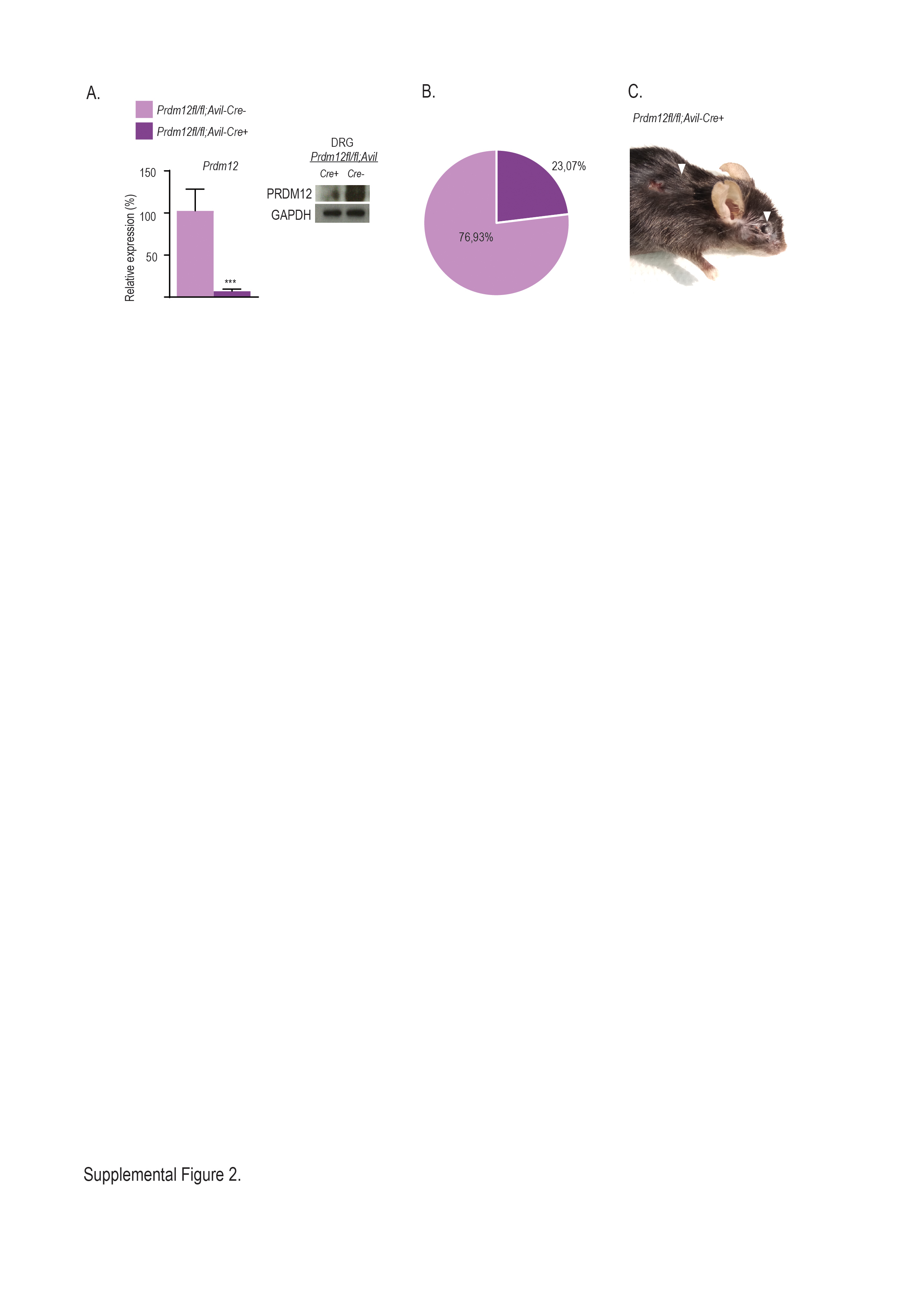

Supplement: Supplementary file 4 [file Image_2.JPEG]

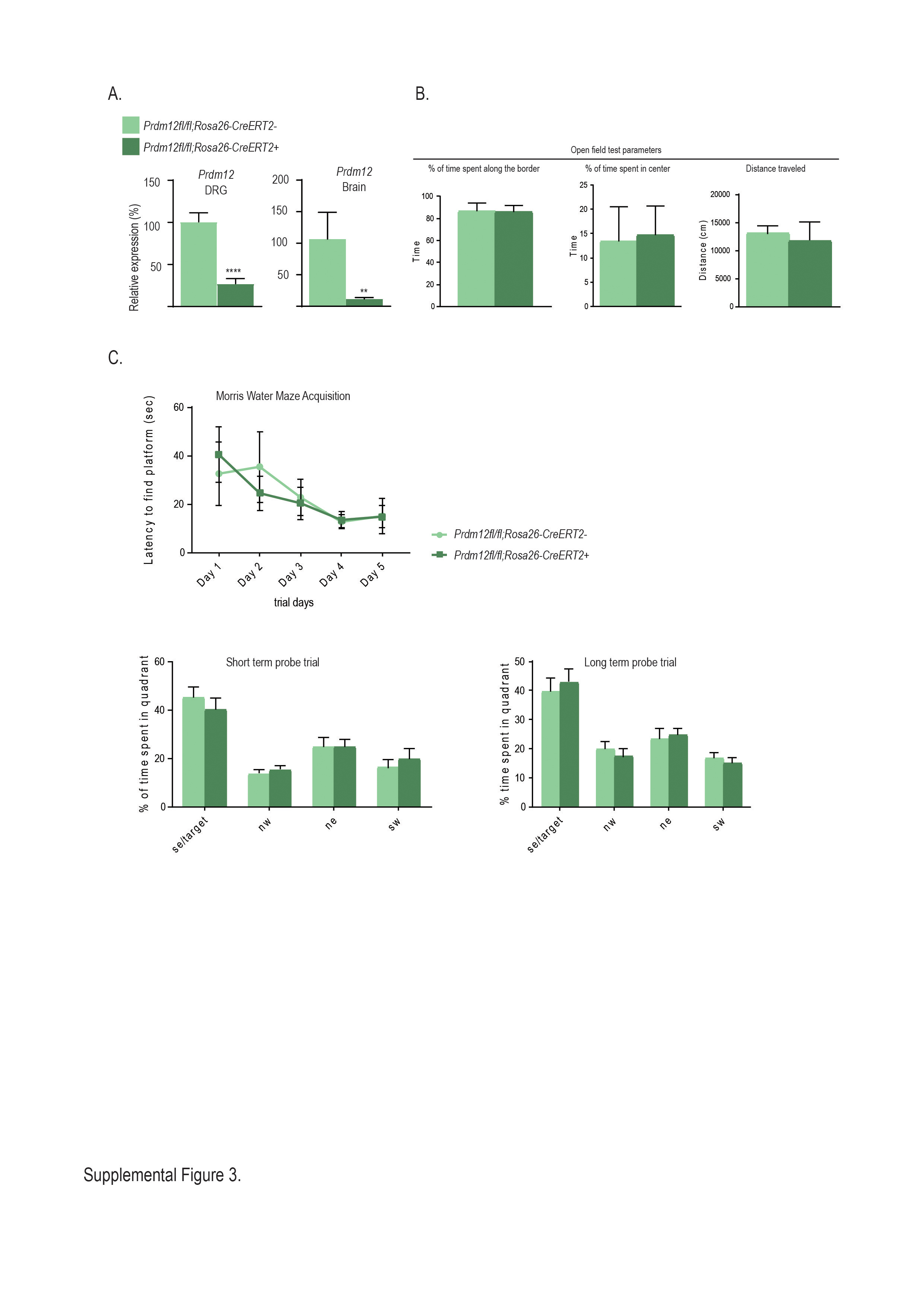

Supplement: Supplementary file 5 [file Image_3.JPEG]

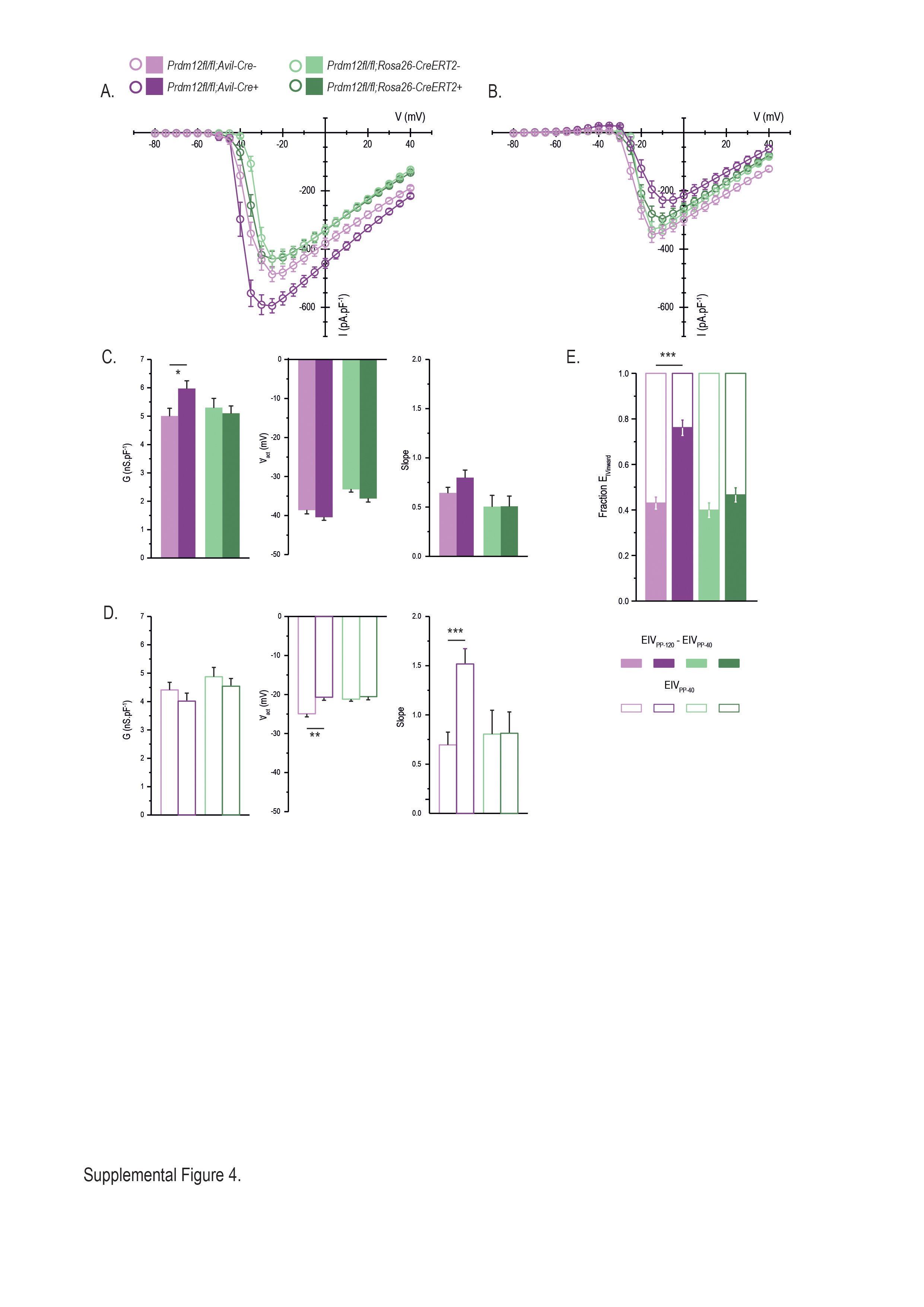

Supplement: Supplementary file 6 [file Image_4.JPEG]

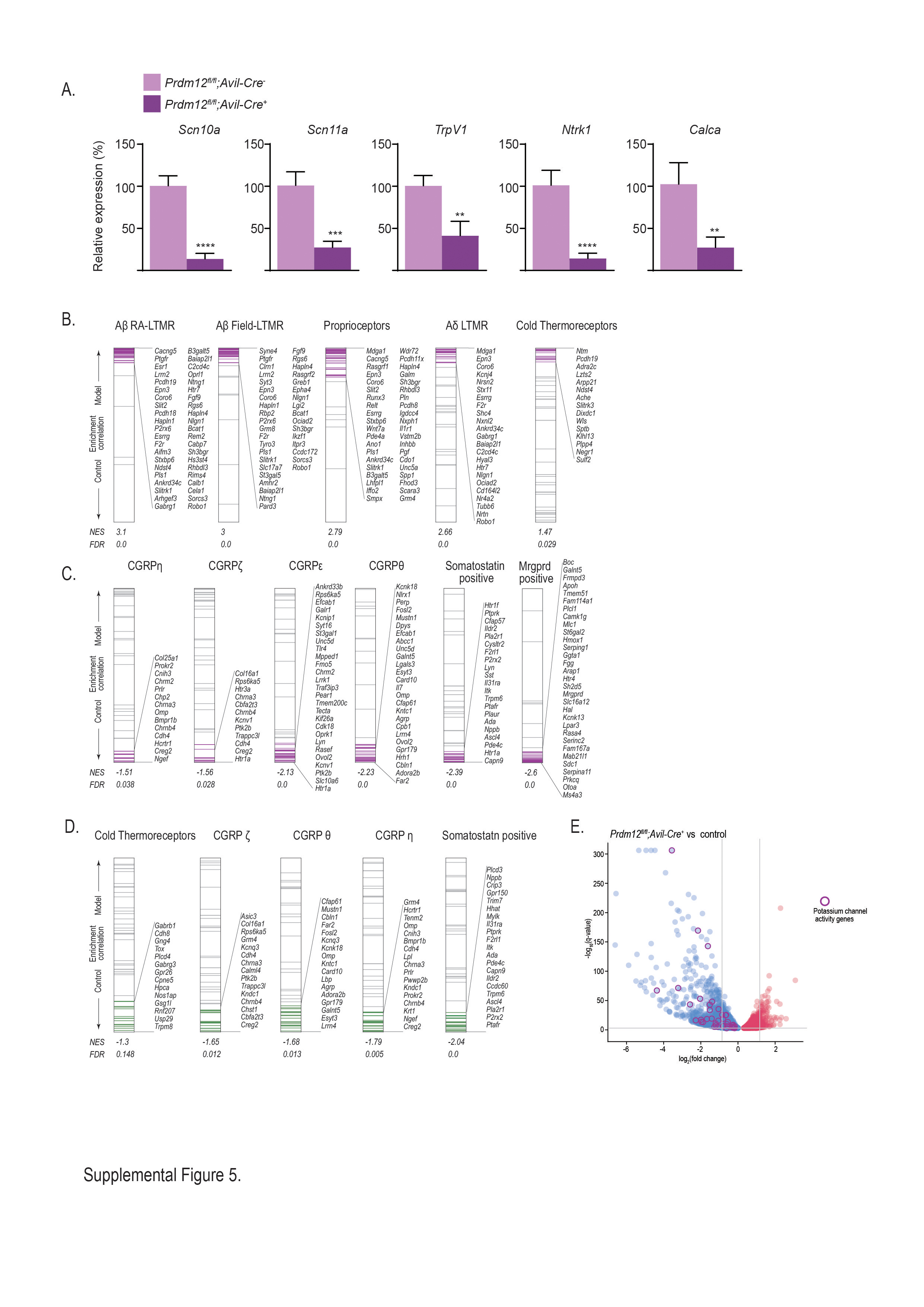

Supplement: Supplementary file 7 [file Image_5.JPEG]
